# Epidemiological characteristics and structural-functional implications of spike protein variations in a bovine coronavirus isolate from diarrheic calves

**DOI:** 10.1080/21505594.2026.2712736

**Published:** 2026-08-17

**Authors:** Fei Teng, Xiaoman Li, Hongzhe Zhao, Yingying Ma, Yingchun Wang, Mingze Chen, Wei Wu, Ting Xiao, Yuxin Wei, Sen Gao, Xinrui Li, Changcheng Zhu, Guiwei Li, Yanping Jiang, Jiaxuan Li, Wen Cui, Xinyuan Qiao

**Affiliations:** a Heilongjiang Key Laboratory for Animal Disease Control and Pharmaceutical Development, Department of Preventive Veterinary, College of Veterinary Medicine, Northeast Agricultural University, Harbin, People’s Republic of China; b Institute of Rural Revitalization Science and Technology, Heilongjiang Academy of Agricultural Sciences, Harbin, China

**Keywords:** Bovine coronavirus, meta-analysis, spike protein, structural modeling, genomic characterization

## Abstract

Bovine coronavirus (BCoV) is an important pathogen associated with enteric disease in calves, contributing to significant economic losses worldwide. To provide an updated overview of BCoV epidemiology, we conducted a systematic review and meta-analysis of studies published up to October 2025. Seventy-two eligible studies comprising 29,045 samples from nine countries were included, yielding a pooled global prevalence of 20.39% (95% CI: 15.07–26.99). Substantial heterogeneity was observed, reflecting variations in geographic regions, detection methods, and study populations. To complement the epidemiological analysis, 298 fecal samples from diarrheic calves in northeastern China were screened by RT-PCR, identifying 36 BCoV-positive samples (12.08%). Co-infection analysis revealed that most positive samples contained additional enteric viruses, indicating complex viral interactions in calf diarrhea. A novel BCoV strain was isolated in MDBK cells and designated DDFX98. Viral identity was confirmed by PCR, transmission electron microscopy, and immunofluorescence assay. Complete genome sequencing demonstrated that DDFX98 belongs to the GIIb subtype and shares high nucleotide identity with contemporary circulating strains. Comparative analysis of the spike (S) protein revealed multiple amino acid substitutions predominantly located within the S1 subunit. Structural modeling suggested localized conformational variations, particularly in surface-exposed and flexible regions, while preserving the overall spike architecture. In silico predictions further indicated minor alterations in glycosylation potential and epitope-associated regions. Collectively, this study integrates global epidemiological evidence with molecular and structural characterization of a contemporary BCoV isolate, providing insights into S protein variability and its potential structural – functional implications.

## Introduction

Bovine coronavirus (BCoV) is an important pathogen associated with enteric and respiratory diseases in cattle, including neonatal calf diarrhea and winter dysentery in adult cattle [1]. BCoV belongs to the genus Betacoronavirus within the family Coronaviridae and is classified in the subgenus Embecovirus. Virions are enveloped and predominantly spherical, with diameters of approximately 80–160 nm. The viral genome is a positive-sense single-stranded RNA of about 32 kb, making BCoV one of the largest known RNA viruses [2].

The BCoV genome encodes five structural proteins: hemagglutinin/esterase (HE), spike (S), envelope (E), membrane (M), and nucleocapsid (N) proteins [3]. Among these, the S protein plays a central role in viral entry and host interaction. The S gene (4092 bp) encodes a 1363-amino-acid glycoprotein that forms the characteristic crown-like projections on the virion surface. After proteolytic cleavage, the S protein is divided into two subunits: S1, which mediates receptor recognition and contains antigenically important regions, and S2, which promotes membrane fusion between viral and host membranes [4]. In addition, BCoV encodes the hemagglutinin-esterase (HE) protein, a feature characteristic of Embecovirus members such as canine respiratory coronavirus (CRCoV), human coronavirus OC43 (HCoV-OC43), turkey coronavirus, and murine coronavirus. The HE protein possesses receptor-destroying esterase activity and may function as a secondary attachment protein during viral infection [5].

BCoV was first identified in the United States in 1972 and has since been reported in China, Canada, Japan, India, Australia, and various European countries. The virus is widely distributed in cattle populations and outbreaks frequently occur during colder seasons. Infection in calves may cause severe diarrhea and mortality, whereas infection in adult cattle is associated with winter dysentery and reduced milk production, resulting in considerable economic losses to the cattle industry. Although BCoV infection is primarily associated with enteric disease, increasing evidence indicates that it also contributes to respiratory infections in cattle [6–9]. Moreover, in intensive farming environments, cattle are continuously exposed to multiple pathogens, making BCoV highly susceptible to co-infection with other viruses, such as bovine viral diarrhea virus (BVDV), bovine enterovirus (BEV), bovine rotavirus (BRV), bovine norovirus (BNoV) and so on [10]. Such co-infections may exacerbate disease severity and complicate clinical diagnosis and control measures.

BCoV is also considered one of the first coronaviruses to cross the species barrier. It not only infects cattle but has also been detected in other animal populations, such as giraffes, sheep, and rodents [11–13]. This broad host adaptability suggests that BCoV has the potential to cross species barriers. Moreover, BCoV shares substantial genetic and antigenic similarity with CRCoV and HCoV-OC43 [14], suggesting possible historical cross-species transmission events.

Despite increasing recognition of the global distribution and evolutionary diversity of BCoV, studies integrating epidemiological analysis with molecular and structural characterization of contemporary strains remain limited. In the present study, we first conducted meta-analysis to evaluate the global prevalence and epidemiological patterns of BCoV infection. We then investigated BCoV circulation in diarrheic calves in northeastern China and successfully isolated a contemporary strain, designated DDFX98. The isolate was further characterized through whole-genome sequencing, phylogenetic analysis, and spike protein – centered structural and functional predictions. These analyses provide an integrated perspective on BCoV epidemiology and molecular evolution and offer insights into the structural implications of spike protein variation in circulating strains.

## Materials and methods

### Meta-analysis methods

To evaluate the prevalence of BCoV infections worldwide, a systematic review was performed on relevant Chinese and English literatures published from database establishment to 1 October 2025. The literatures were retrieved from five databases: China National Knowledge Infrastructure (CNKI), VIP Chinese Journal Database, Wanfang Data, PubMed, and ScienceDirect.Advanced searches were conducted in the three Chinese databases using the keywords “Cattle” and “Bovine Coronavirus.” In ScienceDirect, the keywords “Bovine Coronavirus” and “Cow” were used. In PubMed, the MeSH terms “Coronavirus, Bovine” and “Cattle” and their entry terms were adopted.

During the literature screening phase, studies related to BCoV infection were initially selected based on titles. Then, two reviewers independently screened the literatures, and full-text reviews and data extraction were further performed for eligible studies. The inclusion criteria for this systematic review and meta-analysis were as follows: 1. The research subjects were cattle; 2. The studies clearly included individuals positive for coronavirus infection; 3. The study data were complete, including key information such as the number of infected individuals and total population, number of positive samples, detection location, and sampling location; 4. The articles had full-text availability. This study was conducted and reported in accordance with the Preferred Reporting Items for Systematic Reviews and Meta-Analyses (PRISMA) guidelines. The PRISMA flow diagram illustrating the study selection process is presented in supplementary material, reference number [15].

In this study, the meta package in R software version 4.5.0 was used for data analysis. Prior to conducting the meta-analysis, five data transformation methods were tested to make the data conform more closely to a Gaussian distribution, namely: no transformation (PRAW), logarithmic transformation (PLN), logit transformation (PLOGIT), arcsine transformation (PAS), and double arcsine transformation (PFT). The effectiveness of transformation was determined by the Shapiro-Wilk normality test, with a W-value close to 1 and a *p*-value > 0.05 as the criteria for judging that the data conformed to a Gaussian distribution.

Heterogeneity between studies was calculated using the Cochran-Q test, *I^2^* statistic, and *χ^2^* test. A *p*-value < 0.05 combined with an *I^2^* value of 50% was used to define a statistically significant level of heterogeneity. Based on the heterogeneity results of the included studies, a random-effects model was selected for analysis in this study. A forest plot was used for comprehensive analysis, and funnel plot and Egger’s test were employed to assess publication bias of the studies: when publication bias existed in the included articles, the funnel plot showed an asymmetric distribution with an overall skewed pattern; in the absence of bias, the theoretical value of the regression intercept in Egger’s test was 0. Meanwhile, the stability of individual study results was evaluated via trim and fill analysis and sensitivity analysis.

To further explore the potential sources of heterogeneity, univariate and multivariate models were analyzed to identify the factors influencing heterogeneity. The specific factors analyzed included gender, season, detection method, etc.

### Sample collection and processing

In the spring of 2023–2024, a total of 298 fecal samples were collected from diarrheic calves across multiple farms in Inner Mongolia, Heilongjiang, Jilin, and Liaoning provinces, China. Verbal informed consent was obtained from the owners of the animals prior to sample collection. The farmers-initiated contact following sudden diarrheal outbreaks occurred on their farms, voluntarily submitted samples to our laboratory for analysis. During phone consultations, they were informed of the research objectives and agreed to include the calf diarrhea samples in the study. Written consent was not feasible due to practical constraints such as limited literacy levels in remote areas. The use of verbal consent and the study protocol were reviewed and approved by the Animal Science Ethics Committee of Heilongjiang Province and the Laboratory Animal Ethics Committee of Northeast Agricultural University (Approval No.: NEAUEC20200321). This study was conducted and reported in accordance with the ARRIVE guidelines for animal research. The fecal samples were transported on dry ice to the laboratory, resuspended in 1 mL of PBS, vortexed thoroughly, and centrifuged at 8000 × g for 10 min at 4°C to collect the supernatant. A 200 μL aliquot of the supernatant was used for identification by reverse transcription-PCR (RT-PCR), while the remaining portion was reserved for further analysis.

### RNA extraction and RT-PCR

Total RNA was extracted from the fecal samples using TRIzol reagent (TIANGEN BIOTECH, Beijing, China) following the protocol described in the cited reference [16]. Total RNA was reverse-transcribed using a reverse transcription kit (TaKaRa, Dalian, China). The resulting cDNA obtained from reverse transcription was used as a template for PCR amplification of the N gene of BCoV.

### Viral isolation and identification

Fecal samples (200 mg) from diarrheal calves that tested positive for BCoV by RT-PCR were subjected to virus isolation. Briefly, fecal suspensions were centrifuged at 8000 × g for 10 min, filtered through 0.22 µm pore size filters (Biosharp, Hefei, China) to remove bacteria. After incubation with trypsin (final concentration 30 μg/mL) for 30 min, the suspension was inoculated onto MDBK, HRT-18 and Vero cells. MDBK, HRT-18, and Vero cells were selected because previous studies have reported that these cell lines can support the isolation or replication of bovine coronavirus or other coronaviruses, although their susceptibility may vary among different viral strains [17–19]. Following a 1-h adsorption at 37°C, the medium was replaced with serum-free RPMI 1640 (Thermo Fisher Scientific, Waltham, MA, USA) containing 1 μg/mL trypsin, and the cells were monitored daily. After 96 hours of culture, the virus was harvested, and the cell culture was subjected to three freeze-thaw cycles before being stored at − 80°C for future use.

### Negative staining transmission electron microscopy (TEM)

A 100 mL aliquot of virus-containing supernatant from MDBK cells was centrifuged sequentially at 3000 rpm for 15 min, 5000 rpm for 15 min, and 10,000 rpm for 15 min, after which the viral supernatant was retained. The supernatant was subsequently subjected to ultracentrifugation at 100,000 × g for 4 h using a Beckman Optima MAX-XP (Beckman, USA), and the pellet was resuspended in PBS. A 10 µL aliquot of the viral sample was placed onto a carbon-coated grid for and then stained with 2% phosphotungstic acid for 1 min. The viral morphology was observed using a Tecnai G2 Spirit TWIN transmission electron microscope (Philips, Eindhoven, the Netherlands).

### The immunofluorescence assay (IFA)

BCoV was inoculated into MDBK cells grown to 60–80% confluence in 96-well plates. At 72 h post-inoculation (hpi), the cells were fixed with 4% paraformaldehyde for 30 min, washed three times with PBS, permeabilized with 0.2% Triton X-100 (Beyotime, Shanghai, China) for 10 min, and washed three additional times with PBS. The cells were then blocked with 3% bovine serum albumin (BSA) at 37°C for 30 min. After washing, the cells were incubated with an anti-BCoV monoclonal antibody at 37°C for 1 h, followed by three washes with PBS. The cells were then incubated with goat anti-mouse IgG-FITC (1:200, laboratory stock) in the dark for 30 min, followed by staining with DAPI (1:200, Biosharp, Hefei, China) at room temperature (RT) in the dark for 5 min. Finally, the cells were rinsed with PBS and examined under a ZEISS LSM 900 fluorescence microscope.

### Genome sequencing and phylogeny analysis

Library preparation and high-throughput sequencing were performed by ComateBioscience Co.,Ltd. (Jilin, China). The reference genome sequence of the endemic BCoV strain was obtained from NCBI Genbank (Table S3 and S4). Phylogenetic trees based on the complete genome and S gene sequences were constructed using MEGA11. The maximum likelihood (ML) method was applied with 1,000 bootstrap replicates. The resulting phylogenetic trees were visualized and aesthetically refined using the Interactive Tree of Life (iTOL) platform. Nucleotide sequence identities between the reference strains and DDFX98 were analyzed using the MegAlign program (DNASTAR Lasergene, v7.1.0.44). The resulting homology matrix was subsequently used to generate heatmaps with Origin (OriginPro 2021).

### Bioinformatics and structural analyses of the S protein

The amino acid sequence of the S gene was analyzed using ESPript 3.0 (https://espript.ibcp.fr/ESPript/ESPript/) to predict its secondary structure. The tertiary structure of the S protein was modeled using Swiss-Model (https://swissmodel.expasy.org), an open-access modeling server from the Swiss Institute of Bioinformatics [20,21]. The S protein of human coronavirus OC43 (SMTL ID: 9blk.1) was used as a template for constructing the S-monomer tertiary structure.

Potential N-linked glycosylation sites in mammalian proteins were predicted using NetNGlyc 1.0 (https://services.healthtech.dtu.dk/services/NetNGlyc-1.0/). Protease cleavage sites were predicted using the ProP 1.0 server, and the cutoff value was displayed as a reference line (https://services.healthtech.dtu.dk/services/ProP-1.0/). Transmembrane regions and functional topology of the S protein were analyzed using DeepTMHMM 1.0 (https://services.healthtech.dtu.dk/services/DeepTMHMM-1.0/). B-cell epitopes of the classical strain U00735 were predicted using the immunoinformatics tools BepiPred (https://services.healthtech.dtu.dk/services/BepiPred-2.0/), SVMTriP (http://sysbio.unl.edu/SVMTriP/), and IEDB (http://tools.iedb.org/bcell/). Furthermore, S protein mutations in field isolates were analyzed to assess their potential impact on these predicted epitopes [22–26].

## Results

### Global prevalence of BCoV based on meta-analysis

**Figure 1.**
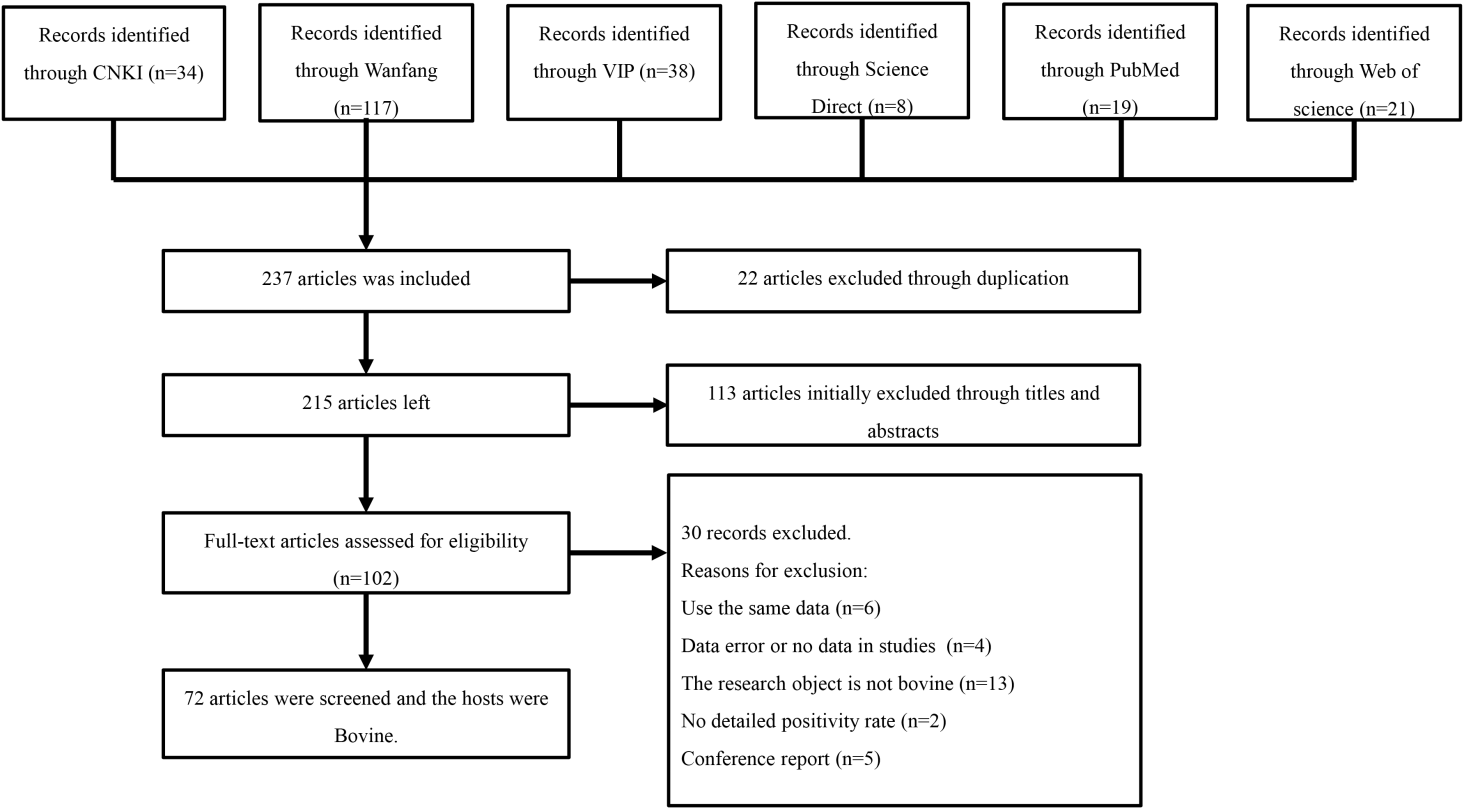
Flow diagram of literature search and selection.

A total of 237 relevant articles were retrieved from five databases in this study. After initial screening and full-text evaluation, ineligible articles were excluded, and finally 72 articles were included for meta-analysis (Figure 1, Table S1). The included studies covered 9 countries, with a total sample size of 29,045 samples, among which 6,095 were BCoV-positive (Table S1). The pooled prevalence was calculated using the Freeman – Tukey double arcsine transformation (PLN), as this transformation produced the highest Shapiro – Wilk W statistic (0.9169) among all evaluated methods, indicating the best approximation to normality (Table S2).

The forest plot of the included studies showed extremely high heterogeneity among all datasets (*χ*^2^ = 2.4320, *I*^2^ = 97.9%, *p* = 0) (Figure S1). The funnel plot showed incompletely symmetrical distribution of data points, suggesting potential publication bias or small-sample bias (Figure S2a). Egger’s regression test indicated significant funnel plot asymmetry (*t* = −11.95, df = 70, *p* < 0.0001; bias estimate = −13.15, SE = 1.10), suggesting the presence of small-study effects and potential publication bias (Figure S2b). Trim-and-fill analysis indicated that 34 studies needed to be supplemented, which altered the pooled effect size (Figure S2c). Sensitivity analysis showed that the pooled results did not change significantly after omitting any single study, with a consistent overall trend (Figure S3), verifying that the results of this analysis were rational and reliable. Figure S4 shows the global distribution map of BCoV prevalence.

Based on the analysis of the included articles, the global pooled prevalence of BCoV infection was 20.39% (95% CI: 15.07–26.99) (Table 1). Among different detection methods, ELISA had the highest positive rate, at 21.86% (95% CI: 12.15–39.34). The prevalence was highest in male cattle, at 31.18% (95% CI: 3.06–100.00). Cattle with diarrheic symptoms had the highest prevalence of 18.33% (95% CI: 12.63–26.61). Among different seasons, the prevalence was highest in spring and winter, both at 19.73%, suggesting that spring and winter may be high-incidence seasons for BCoV infection.

**Table 1.** Summary of global *bovine coronavirus* in bovine infection rates.

						Heterogeneity			Univariate meta-regression	
Variable	Category	No. studies	No. examined	No. positive	% (95% CI*)	*χ*^2^	*p-*value	*I*^2^ (%)	*p-*value*	Coefficient (95% CI)
Testing method	RT-qPCR	14	4877	922	20.28% (12.28–33.49)	2777.31	<0.01	99.4	0.1821	−0.3631(−0.8965 to 0.1702)
	PCR	43	16,967	3616	17.75% (12.38–20.03)	1970.39	<0.01	97.9		
	ELISA	19	7127	1867	21.86% (12.15–39.34)	1300.41	<0.01	99.0		
Gender	Male	2	111	19	31.18% (3.06–100.00)	56.01	<0.01	98.2	0.6442	0.6452(−2.0925 to 3.3828)
	Female	6	583	108	16.34% (4.07- 65.56)	158.25	<0.01	96.8		
Disease	Diarrhea	32	8620	1542	18.33% (12.63–26.61)	1467.40	<0.01	97.9	0.2834	−0.5379(−1.5207 to 0.4449)
	Non-diarrheal	6	1343	226	10.02% (3.38- 29.73)	113.86	<0.01	95.6		
	Respiratory diseases	4	660	106	18.23% (7.01- 47.37)	78.43	<0.01	96.2		
Season	Spring	3	633	210	19.73% (8.20–46.11)	143.43	<0.01	95.4	0.5389	−0.3078(−1.2895 to 0.6740)
	Summer	3	300	35	12.49% (6.82–22.87)	8.06	0.0177	75.2		
	Fall	3	675	95	16.89% (9.22–30.92)	22.08	<0.01	90.9		
	Winter	3	526	148	19.73% (10.32–37.70)	16.87	0.0002	88.1		
Total		72	29,045	6095	20.39% (15.07–26.99)					

CI*: confidence interval; NA: not applicable; *p* value *: *p* < 0.05 is statistically significant.

### Co-infection of BCoV with multiple pathogens in calves with diarrhea

**Figure 2.**
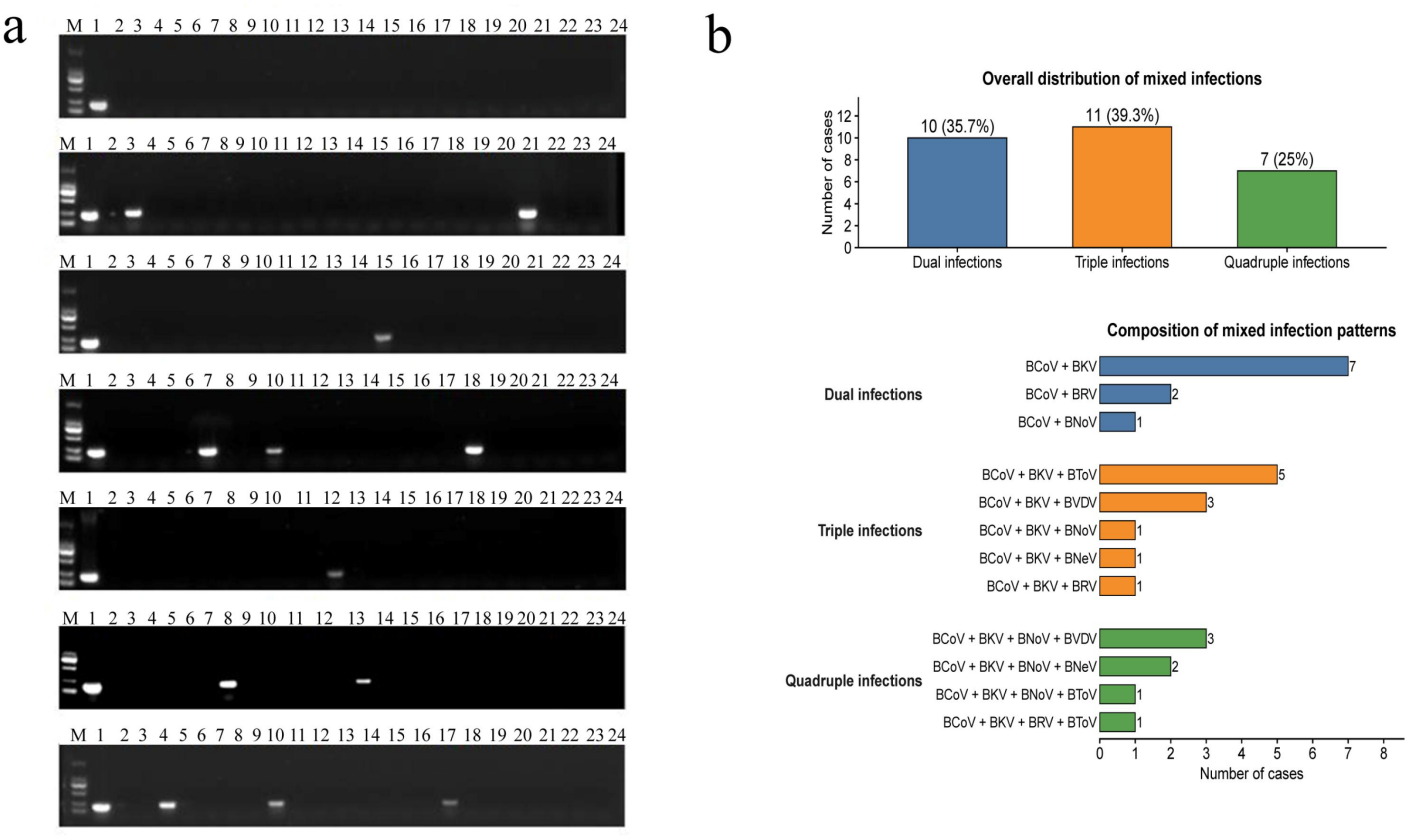
Detection of BCoV and co-infection patterns of enteric viruses in diarrheic calf samples. (a) Detection of BCoV in 298 diarrheic calf samples by PCR. (b) Co-infection patterns among seven diarrhea-associated bovine viruses in 298 diarrheic calf samples.

A total of 298 diarrheic fecal samples were collected from cattle farms in Inner Mongolia, Heilongjiang, Liaoning, and Jilin provinces. After processing, viral RNA was extracted and subjected to RT-PCR using BCoV-specific primers. Specific amplification bands were obtained from 36 samples, which were confirmed as BCoV-positive by sequencing, corresponding to a positivity rate of 12.08% Figure 2(a). In addition, we investigated the infection and co-infection rates of other diarrhea-associated bovine viruses. The pathogens detected by nested PCR included BKV (36.58%, 109/298), BRV (4.70%, 14/298), BNoV (3.69%, 11/298), BToV (6.70%, 20/298), BNeV (6.38%, 19/298), BAstV (2.35%, 7/298), and BVDV (2.68%, 8/298) (Table 2). As shown in Figure 2(b), 77.78% (28/36) of the BCoV-positive samples contained at least two pathogens, and 50% (18/36) harbored three to four different viral agents. Among the BCoV-positive samples, BKV and BToV exhibited the highest co-infection rates, at 19.44% (7/36) and 13.89% (5/36), respectively. These findings indicate that BCoV infection frequently occurs in combination with other enteric viruses, which may exacerbate the severity of diarrhea in calves.

**Table 2.** Prevalence of diarrhea-associated bovine viruses in 298 calf samples.

Farm	BCoV	BKV	BRV	BNoV	BToV	BAstV	BNeV	BVDV
Heilongjiang Farm 1	0	0	25%	0	0	0	0	0
Liaoning Farm 1	0	0	0	0	0	0	0	0
Inner Mongolia Farm 1	16.70%	50%	0	0	16.70%	0	0	0
Heilongjiang Farm 2	1.72%	25%	0	0	0	0	0	1.72%
Inner Mongolia Farm 2	11.11%	44.44%	0	0	0	11.11%	0	0
Inner Mongolia Farm 3	12.50%	4.17%	4.17%	0	0	4.17%	4.17%	0
Jilin Farm 1	1.48%	25.19%	3.70%	0.74%	5.93%	2.96%	1.48%	0
Heilongjiang Farm 3	52.38%	9.52%	14.29%	0	28.27%	4.76%	28.81%	0
Inner Mongolia Farm 4	0	60%	20%	0	6.67%	0	46.67%	0
Heilongjiang Farm 4	80%	95%	0	0	45%	0	15%	40%
Overall prevalence	12.08%	36.58%	4.70%	3.69%	6.70%	2.35%	6.38%	2.68%

### Virus isolation and identification characterization

**Figure 3.**
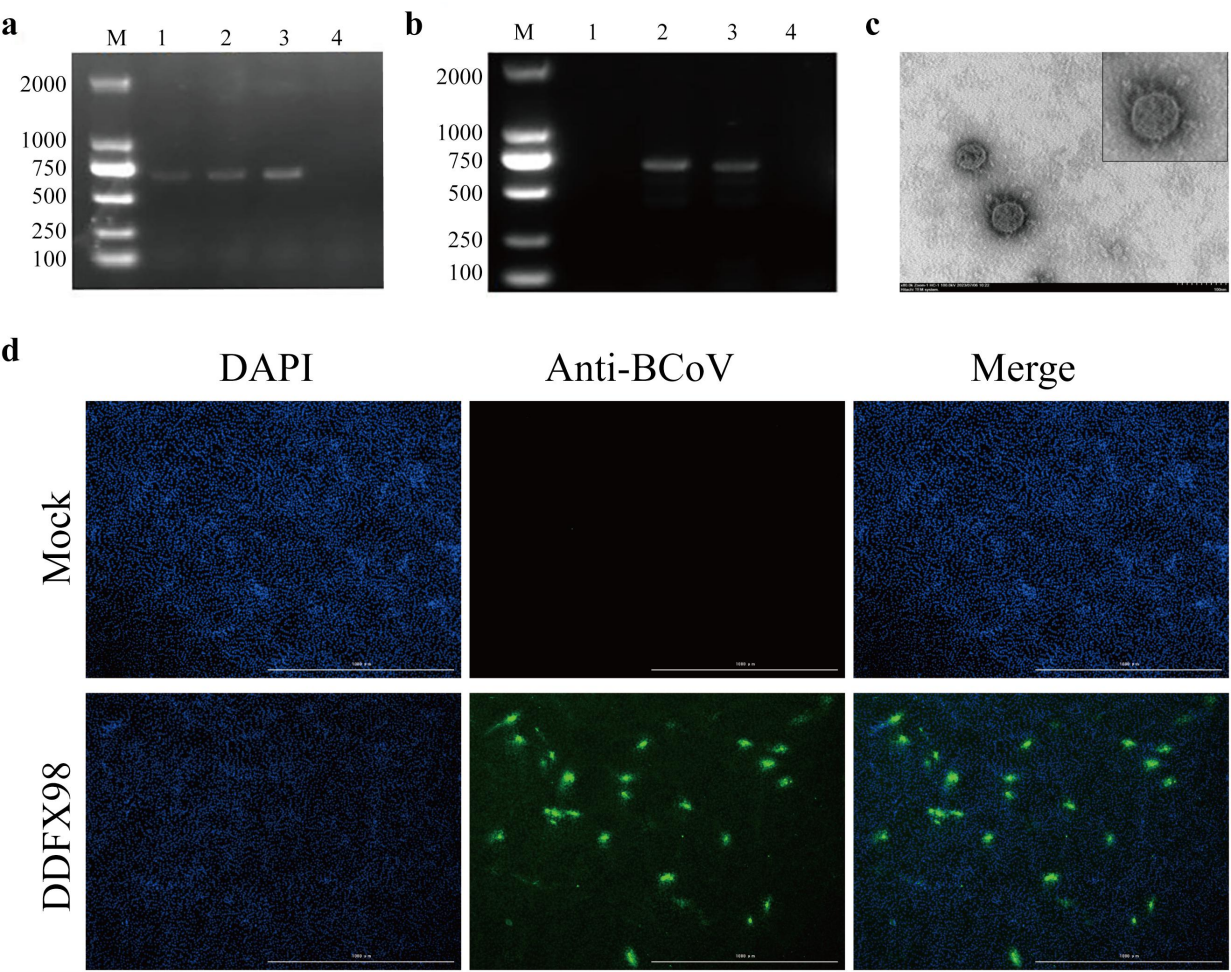
Isolation and characterization of the BCoV strain DDFX98. (a) PCR detection of BCoV following three blind passages in MDBK cells. Lane M, DL2000 DNA marker; lanes 1–3, MDBK cell lysates from blind passages 1–3; lane 4, negative control. (b) PCR detection of BCoV following three blind passages in HRT cells. Lane M, DL2000 DNA marker; lane 1, negative control; lanes 2–4, HRT cell lysates from blind passages 1–3. (c) Transmission electron microscopy of BCoV particles in MDBK cell supernatants. (d) Indirect immunofluorescence assay confirming BCoV infection in MDBK cells.

PCR-positive fecal suspensions were resuspended in PBS, and the clarified supernatants were filtered to remove bacteria before inoculation onto MDBK, HRT-18 and Vero cells. After three blind passages, culture supernatants from each passage were collected and analyzed by PCR. As shown in Figure 3(a), BCoV was detected throughout all three blind passages in MDBK cells. In contrast, only the first two passages were positive in HRT-18 cells Figure 3(b), whereas no BCoV signal was detected in Vero cells across three passages (the results are not shown). Transmission electron microscopy revealed the presence of typical coronavirus-like particles in the supernatants of BCoV-infected MDBK cells, with diameters of approximately 160–220 nm Figure 3(c). The presence of BCoV was further confirmed by indirect immunofluorescence assay (IFA) using a polyclonal antibody against the BCoV nucleocapsid (N) protein Figure 3(d).

Together, these results demonstrate a BCoV strain was successfully isolated in this study, designated DDFX98.

### Genome sequencing and phylogeny analysis

To characterize the genomic features of the BCoV isolate obtained in this study, the complete genome of strain DDFX98 was sequenced and deposited in the GenBank database under accession number PX984620. The genome comprised 31,012 nucleotides (nt). The lengths of the major coding regions were as follows: ORF1ab (21,269 nt), HE (1,275 nt), S (4,092 nt), E (255 nt), M (693 nt), and N (1,347 nt).

**Figure 4.**
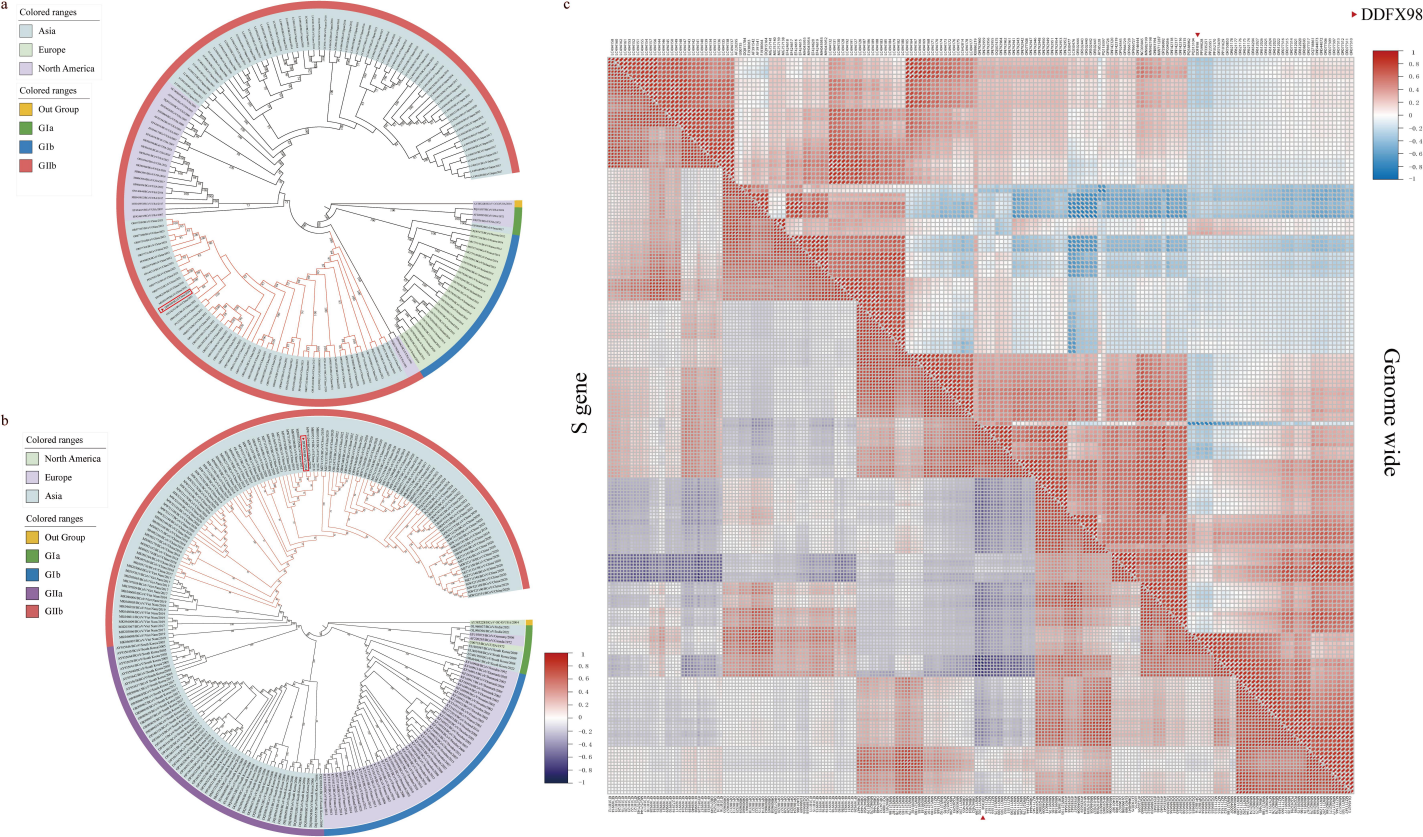
Phylogenetic analysis of the complete genome and S gene of the DDFX98 strain. (a) Whole-genome phylogenetic analysis of DDFX98 and 175 representative BCoV strains from different geographic regions, showing classification into three subtypes (GIa, GIb, and GIIb). The DDFX98 isolate is highlighted by a red box, and the contemporary Chinese lineage is highlighted by red branches. The geographic origin of each strain is indicated by the outer color strip. The branches highlighted in red represent the clade comprising Chinese isolates. (b) S gene-based phylogenetic analysis of DDFX98 and 235 representative BCoV strains from diverse geographic origins, indicating four subtypes (GIa, GIb, GIIa, and GIIb). The DDFX98 isolate is highlighted by a red box, and the contemporary Chinese lineage is highlighted by red branches. The geographic origin of each strain is indicated by the outer color strip. The branches highlighted in red represent the clade comprising Chinese isolates. (c) Normalized nucleotide identity heatmaps based on the complete genome and S gene sequences of BCoV strains.

To investigate its evolutionary placement, the full-length genome sequence of DDFX98 was aligned with 175 representative BCoV strains originating from diverse geographic regions using MEGA software. Phylogenetic analysis based on the complete genome indicated that contemporary BCoV strains are predominantly distributed across Asia, Europe, and North America Figure 4(a). The DDFX98 isolate (indicated by a red triangle) clustered within the GIIb subtype and grouped closely with the Chinese strain ON142315, forming a shared lineage within the China-associated branch. Notably, U.S. strains exhibited closer genetic relatedness to Asian strains and were likewise assigned to the GIIb subtype, whereas European strains were more distantly related and belonged to the GIb subtype. After excluding outgroup sequences, DDFX98 displayed high nucleotide identity with currently circulating BCoV strains, ranging from 97.7% to 99.1%. The highest identity was observed with strain OR621174/China/2022 (99.1%), supporting its classification within a contemporary circulating lineage (Table S5).

Consistent findings were obtained from phylogenetic analysis of the S gene. The S gene sequence of DDFX98 (red triangle) was aligned with 235 representative BCoV S gene sequences Figure 4(b). A well-supported monophyletic clade comprising Korean strains (2005–2022) was observed, which was clearly separated from the GIIb lineage containing recent Chinese and Vietnamese isolates. Nucleotide identity analysis revealed that the DDFX98 S gene shared 96.9%–99.5% identity with reference strains, with the highest similarity to strain MW711294/China/2020 (99.5%) (Table S6). Normalized homology heatmaps based on both the complete genome and S gene further illustrated distinct clustering patterns among strains Figure 4(c).

### Structural characterization of the S protein

The S protein of coronaviruses is located on the outermost surface of the virion and plays a critical role in viral entry and host interaction. Due to its functional importance, the S protein is considered highly susceptible to genetic variation. To further characterize the structural features of the S protein of the DDFX98 isolate, six representative BCoV strains spanning different decades and geographic lineages were selected for comparative analysis. These included the classical prototype strain Mebus (U00735/USA/1972), European strains from the 1990s (KF169908/Sweden/1992) and 2010s (MG757144/France/2014), a Southeast Asian strain (MH203067/VietNam/2017), an East Asian strain (MW711294/China/2020), and a recent European strain (OR271252/Ireland/2022).

**Figure 5.**
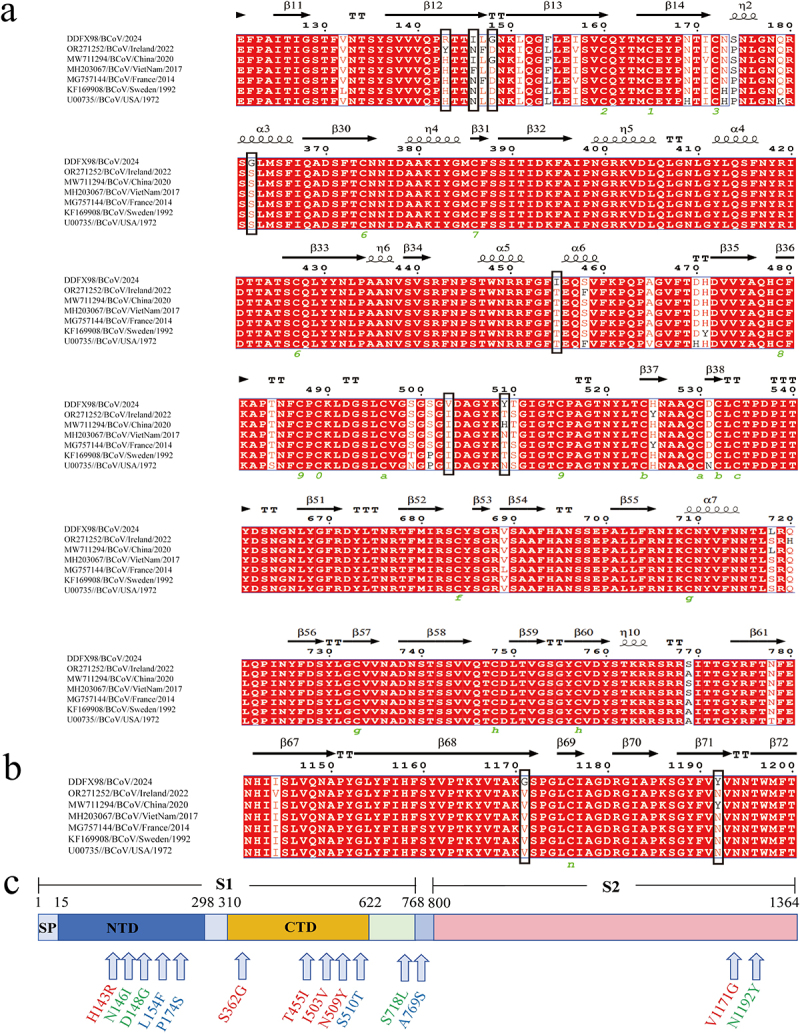
Comparative structural analysis of the S protein of the DDFX98 isolate and six representative BCoV strains. (a) Multiple sequence alignment of the S1 subunit showing the predicted secondary structural elements and amino acid substitution. (b) Multiple sequence alignment of the S1 subunit showing the predicted secondary structural elements and amino acid substitutions. (c) Schematic representation of the unique amino acid substitutions identified in the DDFX98 S protein. Amino acid substitutions are color-coded as follows: residues unique to DDFX98 are shown in red, substitutions shared with contemporary Chinese isolates are shown in green, and substitutions commonly observed among Asian isolates are shown in blue.

Amino acid sequence alignment revealed that the DDFX98 S protein contained no insertions or deletions relative to the reference strains but exhibited multiple amino acid substitutions. Mutation mapping indicated that sequence variation was predominantly concentrated within the S1 subunit (residues 1–768). Among the total mutations, five substitutions (H143R, S362G, T455I, I503V, and N509Y) were uniquely found in the DDFX98 isolate. Three substitutions (N146I, D148G, and S718L) were shared with contemporary Chinese isolates, whereas three substitutions (L154F, P174S, and S510T) were commonly observed among broader Asian isolates Figure 5(a). In contrast, fewer substitutions were observed in the S2 subunit (residues 800–1364). Only one strain-specific substitution (V1171G) and one substitution shared with contemporary Chinese isolates (N1192Y) were identified in this region. In addition, a substitution near the S1/S2 cleavage site (A769S), which is commonly observed among Asian isolates, was also detected Figure 5(b). The spatial distribution of these substitutions within the S protein structure is illustrated in Figure 5(c).

**Figure 6.**
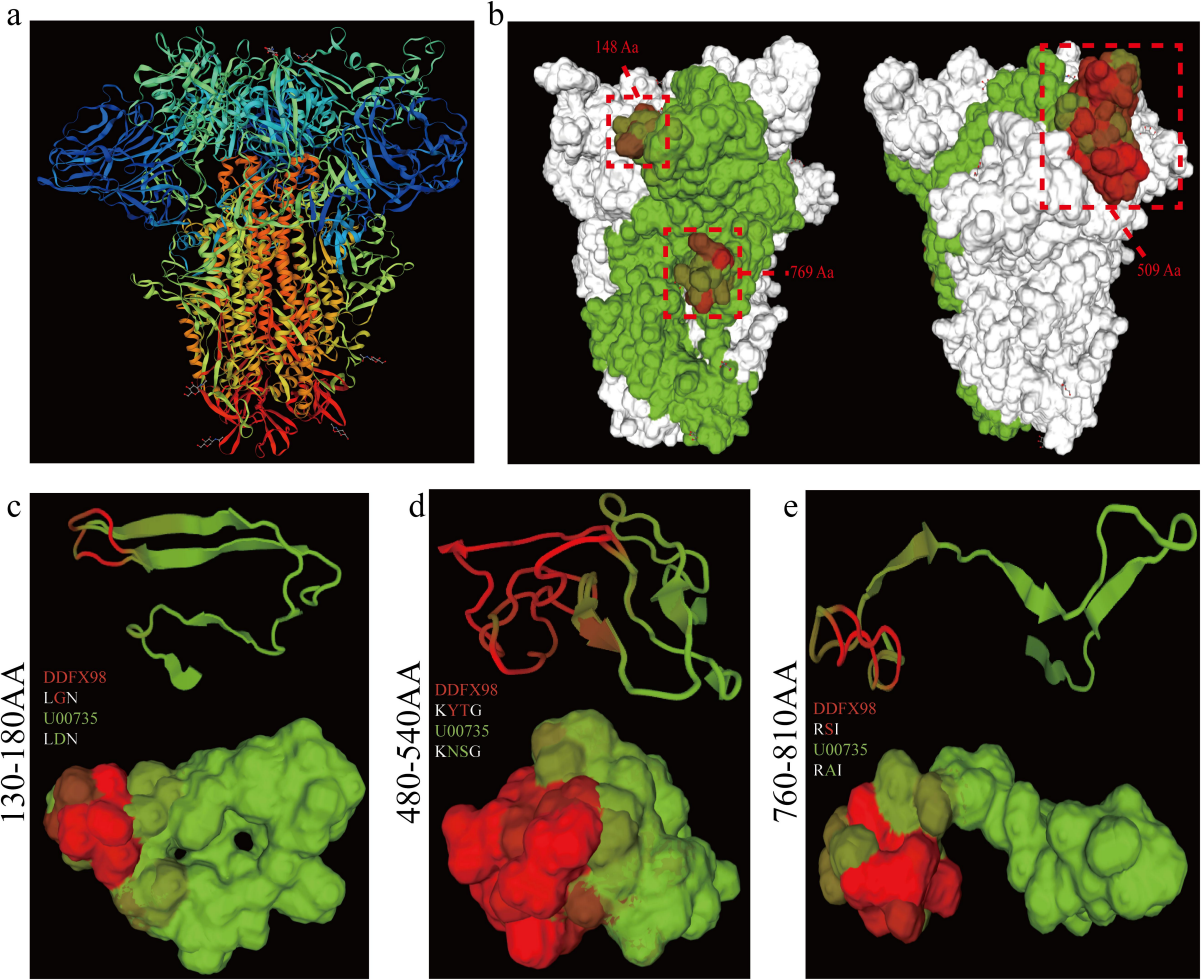
Structural modeling and conformational comparison of the DDFX98 S protein. (a) Predicted three-dimensional structure of the DDFX98 S protein based on SWISS-MODEL using human coronavirus OC43 (SMTL ID: 9blk.1) as the template. (b) Structural superposition of the DDFX98 and reference strain U00735 spike proteins. Regions exhibiting localized conformational differences are highlighted by red dashed boxes, corresponding to residues 148, 509, and 769. (c – e) enlarged views of the three regions showing localized conformational differences surrounding residues 148, 509, and 769, respectively. Ribbon and surface representations are shown to illustrate the structural changes associated with the corresponding amino acid substitutions. Throughout the figure, the DDFX98 structure is shown in red, whereas the U00735 structure is shown in gree.

To assess the potential structural consequences of the observed amino acid substitutions, the three-dimensional structure of the DDFX98 S protein was predicted using the SWISS-MODEL server Figure 6(a). The resulting model was subsequently compared with that of the classical prototype strain Mebus (U00735). Structural superposition of the predicted S protein models revealed three localized conformational differences between DDFX98 and U00735 at positions 148, 509, and 769 Figure 6(b). These substitutions were associated with subtle structural alterations in the N-terminal domain (NTD), the C-terminal domain (CTD) of the S1 subunit, and the S1/S2 junction region.

Residue 148 is located within the NTD of the S1 subunit. The substitution at this position was associated with a minor adjustment in the adjacent turn/loop region near a β-sheet, without disrupting the overall secondary structure Figure 6(c). Residue 509 is positioned within the CTD of the S1 subunit. The mutation at this site resulted primarily in a positional shift of a surface-exposed loop proximal to a β-sheet, leading to a discernible local surface variation in the tertiary structure model Figure 6(d). Residue 769 is located near the S1/S2 junction, adjacent to the KRRSRR cleavage motif (residues 763–768). A localized loop rearrangement was observed in this region in the DDFX98 structural model Figure 6(e).

### Predicted effects of S protein mutations on functional sites

**Figure 7.**
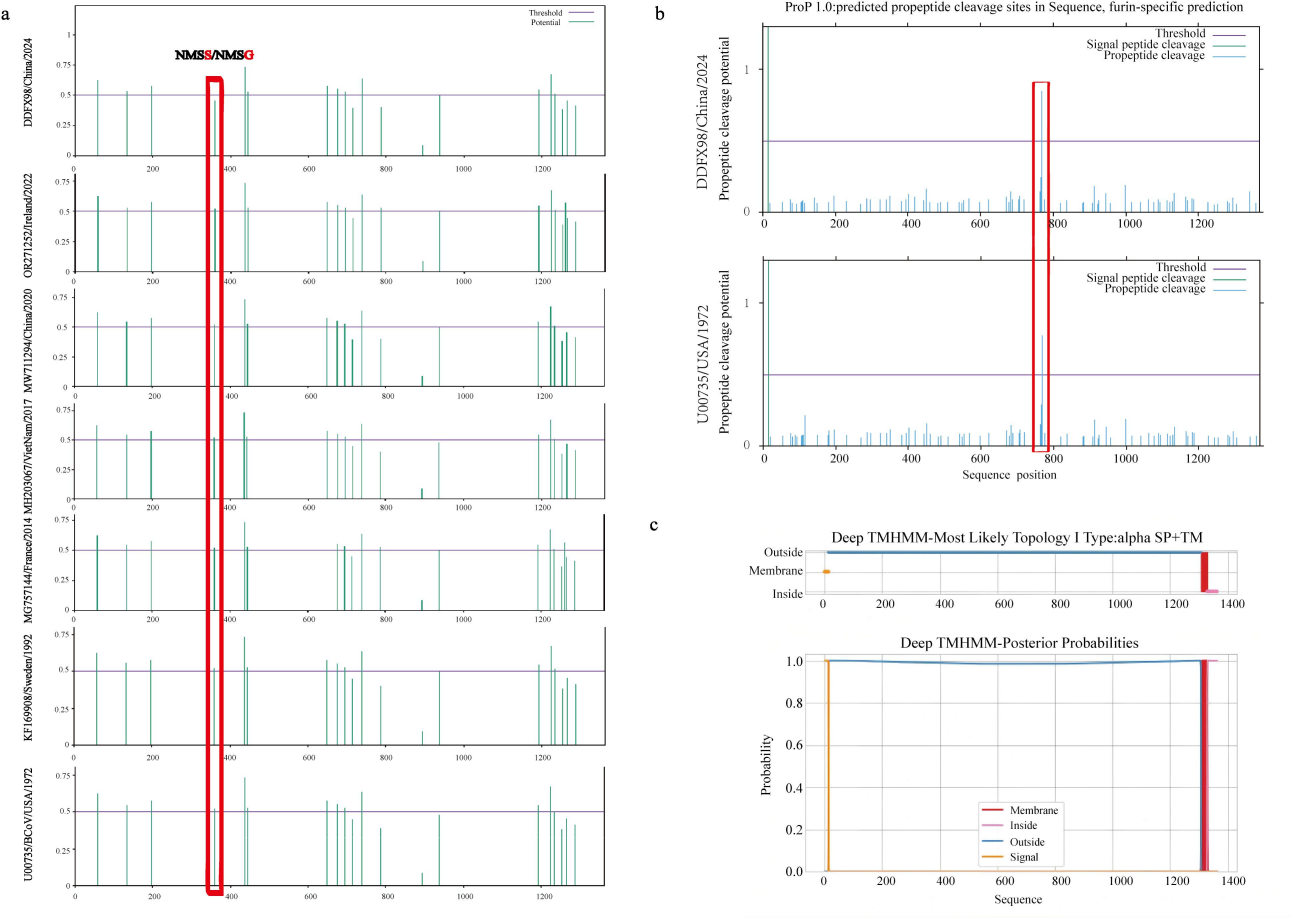
Effects of amino acid substitutions on functional features of the DDFX98 S protein. (a) Prediction of potential N-linked glycosylation sites in the S proteins of DDFX98 and representative BCoV strains using the NetNGlyc server. The red box highlights the glycosylation site affected by the S362G substitution. (b) Prediction of the S1/S2 furin cleavage site in the DDFX98 and reference strain U00735 S proteins using ProP 1.0. The red box indicates the predicted furin cleavage region surrounding the A769S substitution. (c) Predicted transmembrane topology of the DDFX98 S protein generated using DeepTMHMM. The predicted transmembrane region is shown together with the corresponding topology probabilities.

To further evaluate the functional and antigenic characteristics of the S protein, a comprehensive in silico comparison was conducted between DDFX98 and the classical reference strain U00735. Prediction using the NetNGlyc 1.0 server indicated that amino acid substitutions in DDFX98 resulted in the loss of one potential N-linked glycosylation site Figure 7(a).

Furin cleavage site prediction using the ProP 1.0 server revealed prominent cleavage peaks around residue 770 in both U00735 and DDFX98. Despite minor variations in peak intensity, the predicted substitutions did not markedly alter the furin-like protease recognition profile Figure 7(b). Similarly, analysis using DeepTMHMM suggested that the observed substitutions did not affect the predicted transmembrane topology of the S protein Figure 7(c).

B-cell epitope prediction was performed using three independent immunoinformatics tools (BepiPred, SVMTriP, and IEDB). The major antigenic regions identified by these methods were integrated to define the predicted B-cell epitopes of the U00735 S protein (Table 3). Sequence comparison demonstrated that five substitutions (K115D, H142R, N146I, D148G, and S362G) were located within the predicted epitope regions of DDFX98. Notably, three substitutions were clustered within the AA135–150 region. Structural mapping suggested that these substitutions coincide with localized conformational adjustments Figure 5(a) and 6(c). Collectively, these findings suggest that the antigenic landscape of the DDFX98 S protein may differ subtly from that of the classical strain.

**Table 3.** B-cell antigenic epitope of the classical wild-type strain (U00735) S protein.

B-cell antigenic epitope	Amino acid position	B-cell antigenic epitope	Amino acid position
NRFGAISSSLQEIL	1062–1075	IRSCYSGRVSAAFHANSSEP	681–700
DCNKVSSRSAIED	906–918	VSQQLSDSTLVKFSAAQAMEKVNECVKSQSSRINFCGNG	1102–1140
FMSEIKCKTLSIAPSTGV	290–307	QLQVANSLMNGV	866–877
LVKIQAVVNANAEALNNLLQ	1039–1058	NSTSSVVQTC	739–748
TKVIKKGVMYSEFPAITIGS	110–129	NFNMSSLMS	357–365
LGWSVDSCLQGDRCNIFANF	585–604	SYSVVVQPHTTNLD	135–148

## Discussion

BCoV, a member of the genus Betacoronavirus, is a major pathogen causing enteric and respiratory diseases in cattle and is widely associated with neonatal calf diarrhea, winter dysentery in adult cattle, and bovine respiratory disease complex [4]. Since the classical prototype Mebus strain was first described in the United States in 1972, BCoV has spread worldwide and diversified into multiple genetic lineages across different geographical regions. In the present study, phylogenetic analyses based on the whole genome and S gene showed that the DDFX98 isolate belonged to the GIIb lineage and clustered closely with contemporary Chinese/Vietnamese strains. Notably, Kim et al. proposed a phylogenetic classification framework based on the S and HE genes, dividing BCoV strains into classical, Asia/USA, and European lineages. In their study, Korean BCoV strains were considered to have originated from the Asia/USA lineage but subsequently diverged into a distinct monophyletic cluster through independent regional evolution. Consistent with these findings, Korean strains in our phylogenetic tree were clearly separated from the contemporary Chinese/Vietnamese GIIb lineage [27]. This geographic clustering suggests ongoing regional diversification of BCoV in Asia and underscores the need for continuous molecular surveillance to monitor the emergence of genetically distinct regional variants [7,28]. Moreover, the potential zoonotic implications of bovine-like coronaviruses have been discussed, particularly in relation to the evolutionary links between BCoV and certain human coronaviruses [29]. Against this background, comprehensive genomic characterization of emerging BCoV strains is important for monitoring viral evolution, evaluating genetic diversity, and supporting molecular surveillance. In this study, we integrated meta-analysis, virus isolation, and bioinformatic approaches to investigate the epidemiological and molecular characteristics of BCoV. Based on a global overview of BCoV epidemiology, we successfully isolated a contemporary BCoV strain from diarrheic calves in northeastern China, designated DDFX98. This isolate was further characterized by whole-genome sequencing, phylogenetic analysis, and S protein – based structural and functional predictions, providing insights into its genetic characteristics and phylogenetic relationship with contemporary BCoV strains.

Through multi-database retrieval and screening, this study finally included 72 studies from 9 countries involving 29,045 samples for Meta-analysis, systematically estimating the global infection rate of BCoV and providing evidence-based support for the analysis of its epidemiological characteristics and the formulation of prevention and control strategies. The results showed that the global overall infection rate of BCoV was 20.39% (95% CI: 15.07–26.99), indicating that BCoV is widely prevalent in cattle herds worldwide and is one of the important pathogens threatening cattle health [30]. In the subgroup analysis, the infection rate of bulls was 31.18%, which was significantly higher than that of females (16.34%), presumably associated with differences in the physiological characteristics, activity range, and immune status of bulls. The infection rate of cattle with diarrhea symptoms was 18.33%, confirming the pathogenic characteristic of BCoV that invades the digestive tract and causes diarrhea [31]. In the seasonal subgroup, the infection rates in spring and winter both reached 19.73%, which were significantly higher than those in other seasons, suggesting that these two seasons may be the high-incidence periods of BCoV. This is presumably associated with drastic temperature changes during seasonal alternation, decreased immunity of cattle herds, and increased transmission efficiency of the virus in low-temperature environments [32]. From the above Meta-analysis results, we clearly found that seasonal factors and diarrhea symptoms in cattle are both key variables affecting the prevalence rate of BCoV. In addition, a Meta-analysis on the prevalence of BCoV across China conducted by Geng et al. has clearly pointed out that the prevalence rate of BCoV in northeastern China is significantly higher than that in other regions of the country [8], indicating that this region is a key area with a high risk of BCoV prevalence. Based on the above findings, our study specifically collected fecal samples from cattle with typical diarrhea symptoms in northeastern China in spring, aiming to fill the gap in research on spring BCoV epidemic strains in northeastern China.

BCoV frequently occurs in co-infections with other enteric pathogens, including parasites, bacteria, and viruses, which may collectively contribute to diarrhea [33]. Previous studies have demonstrated significant interactions between bovine viral diarrhea virus (BVDV) and BCoV during the progression of bovine respiratory disease (BRD), where BVDV-induced immunosuppression and impaired host barrier function are thought to facilitate BCoV dissemination and exacerbate tissue damage [34]. In addition, Brunauer et al. reported that BCoV was frequently detected together with bovine rotavirus (BRV), Escherichia coli K99, and Cryptosporidium in fecal samples from calves younger than 60 days, and the presence of BRV significantly increased the likelihood of BCoV detection in diarrheic calves [35]. Consistent with these findings, co-infection with other viruses was observed in 28 of the 36 BCoV-positive samples in this study (77.8%). Among these, 10 samples showed dual infections, 11 exhibited triple infections, and 7 harbored four viral pathogens simultaneously. Mixed infections may also prolong pathogen shedding, increase viral transmission within herds, and complicate the clinical presentation, making etiological diagnosis and disease control more challenging. Moreover, simultaneous infection with multiple enteric viruses may interfere with host innate immune responses and intestinal barrier integrity, thereby creating a more favorable environment for pathogen persistence and disease progression [36,37]. Collectively, these findings further emphasize that comprehensive pathogen surveillance rather than single-pathogen detection is essential for improving the diagnosis, prevention, and control of calf diarrhea under field conditions.

The S protein is the key structural component of coronaviruses and plays a central role in determining viral tropism and infectivity [33]. During viral entry, the S protein is cleaved by host furin-like proteases between residues 768 and 769, generating two functionally distinct subunits, S1 and S2 [38]. The S1 subunit mediates receptor binding and viral attachment, whereas the S2 subunit facilitates membrane fusion between viral and host membranes, enabling delivery of the viral genome into host cells. Genomic analysis of the S protein of the DDFX98 isolate identified six substitutions unique to this strain (H143R, S362G, T455I, I503V, N509Y, and V1171G), three substitutions shared with contemporary Chinese strains (N146I, D148G, and S718L), and four substitutions commonly observed among Asian strains (L154F, P174S, S510T, and A769S) Figure 5(c). Several substitutions were shared with contemporary Chinese and other Asian BCoV strains, suggesting ongoing regional evolution rather than isolate-specific genetic variation. These findings underscore the importance of continuous molecular surveillance to monitor the genetic diversity of circulating BCoV strains. Their potential implications for antigenic variation and vaccine effectiveness warrant further investigation. Structural mapping indicated that most amino acid substitutions were concentrated within the S1 subunit, whereas the S2 subunit remained relatively conserved. This mutation distribution likely reflects functional constraints, as the S1 region contains receptor-binding and antigenically exposed domains that are more susceptible to host immune selection [39,40]. Notably, one substitution (A769S) was located adjacent to the S1/S2 cleavage motif KRRSRR, a critical region required for spike activation by host furin-like proteases and subsequent membrane fusion during viral entry. Structural superposition with the classical Mebus strain revealed localized conformational alterations in this region. Although the alanine-to-serine substitution represents a relatively conservative physicochemical change, the introduction of a hydroxyl group increases local polarity and hydrogen-bonding potential, which may subtly alter the surrounding hydrogen-bonding network and could potentially have implications for the structural environment surrounding the predicted furin cleavage site.

Viral receptors play a critical role in infection by mediating viral recognition, attachment, and entry into host cells. The S1 subunit of the BCoV S protein contains two receptor-binding domains: the N-terminal domain (NTD) and the C-terminal domain (CTD). In general, the NTD interacts with glycan receptors, and multiple studies have demonstrated that sialic acids function as attachment receptors that bind to the NTD [3,41,42]. In contrast, the CTD is typically responsible for binding protein receptors and mediating viral entry. Previous studies have suggested that HLA class I molecules may function as potential receptors for BCoV [14,15]. Local conformational variations within receptor-binding domains may influence host adaptation and viral entry efficiency and may also affect antigenicity and immunogenicity [43]. In the present study, structural comparison identified two regions with conformational differences within the NTD and CTD domains, corresponding to residues D148G and N509Y/S510T. The D148G substitution is located within the S1 NTD and lies in proximity to the predicted sialic acid-binding region, suggesting that this mutation may influence interactions between the S protein and glycan receptors, thereby potentially affecting viral attachment to host cells [44,45]. In addition, two adjacent substitutions, N509Y and S510T, were located within the CTD domain. The N509Y substitution replaces a polar asparagine with a bulkier aromatic tyrosine residue, which may increase local hydrophobicity and steric occupancy, whereas S510T may subtly alter the local hydrogen-bonding network. Together, these substitutions may account for the local surface variation observed in the structural model Figure 6(d). Further bioinformatic analyses suggested that the S362G substitution may alter a potential glycosylation site and predicted antigenic epitopes on the S protein surface. In addition, the substitutions H143R, S362G, N146I, and D148G were all located within predicted antigenic epitope regions (Figure 7 and Table 3). These observations suggest that the identified amino acid substitutions may contribute to structural or antigenic variation of the S protein, although the underlying mechanisms require further investigation.

Despite providing comprehensive epidemiological and molecular insights into contemporary BCoV, several limitations should be acknowledged. The pooled prevalence estimate (20.39%) should be interpreted within the context of the included studies rather than as a universally applicable estimate. None of the investigated covariates reached statistical significance in the meta-regression analysis, and substantial heterogeneity (*I^2^* > 95%) persisted across most subgroups, suggesting that the observed between-study variation may reflect unreported study-level factors rather than differences in detection methods, clinical presentation, or season alone. Importantly, this high heterogeneity itself highlights the need for more standardized reporting, including sampling strategies, case information, and sequencing data, in future BCoV prevalence studies. In addition, although structural modeling and antigenicity analyses provided valuable insights into the potential biological significance of the identified spike protein mutations, these findings remain prediction-based and should be interpreted with caution. Future studies using receptor-binding assays, reverse genetics, neutralization assays, and viral infectivity experiments are needed to validate these predicted functional implications.

In summary, this study conducted a meta-analysis to characterize the global prevalence and epidemiological patterns of BCoV, revealing that seasonal factors and diarrheic symptoms in cattle are key variables influencing BCoV prevalence. The results also identified northeastern China as a region with an elevated risk of BCoV circulation. A contemporary BCoV strain, designated DDFX98, was successfully isolated from this region and further characterized through molecular, phylogenetic, and S protein structural analyses. Multiple amino acid substitutions were identified within the NTD, CTD, and the region adjacent to the S1/S2 cleavage site, which were associated with local conformational changes in the S protein. These findings highlight the ongoing genetic evolution of BCoV and its potential biological consequences. Collectively, this study provides valuable insights for future BCoV surveillance, outbreak early warning, and vaccine development.

## Supplementary Material

Table S3.docx

Table S6.xlsx

Fig S2.png

Table S4.docx

Fig S4.png

Clean Copy of Supplementary Figure and Table Legends - QVIR-2026-0276.R1.docx

Table S5.xlsx

Fig S3.png

Fig S1.png

Table S1.docx

Table S2.docx

## Data Availability

The data of complete CDS region nucleotide sequence of the isolated BCoV strain has been deposited in GenBank under accession number: PX984620. They can be found below: https://www.ncbi.nlm.nih.gov/nuccore/PX984620/. Both the supplementary materials and the raw data supporting this study are available in Mendeley Data (https://doi.org/10.17632/ndk6hs7zky), https://doi.org/10.17632/ndk6hs7zky, reference number [46]. These data were derived from the following resources available in the public domain: NCBI GenBank (https://www.ncbi.nlm.nih.gov/genbank/), China National Knowledge Infrastructure (CNKI) (https://www.cnki.net/), VIP Chinese Journal Database (https://www.cqvip.com/), Wanfang Data (https://www.wanfangdata.com.cn/), PubMed (https://pubmed.ncbi.nlm.nih.gov/), and ScienceDirect (https://www.sciencedirect.com/).
