# Supplementary material for: Epidemiological characteristics and structural-functional implications of spike protein variations in a bovine coronavirus isolate from diarrheic calves
Source: Virulence. 2026 Aug 17;17(1):2712736. doi: 10.1080/21505594.2026.2712736 (PMC13483470; doi:10.1080/21505594.2026.2712736)
Supplement: Supplementary file 1 [file KVIR_A_2712736_SM3659.docx]

**Table S2** Normal distribution tests for normal rates and different transitions of articles hosted by bovine.

| **Conversion form** | **W** | **P** |
| --- | --- | --- |
| PRAW | 0.80108 | 1.834e-08 |
| PLN | 0.91688 | 0.0001559 |
| PLOGIT | Na | NA |
| PAS | 0.87605 | 3.807e-06 |
| PFT | 0.8704 | 2.405e-06 |

"PRAW": raw exchange rate; PLN: log conversion. "PLOGIT": logit transformation; "PAS": arcsine transformation; "PFT": double arcsine transformation;
