# Supplementary material for: Epidemiological characteristics and structural-functional implications of spike protein variations in a bovine coronavirus isolate from diarrheic calves
Source: Virulence. 2026 Aug 17;17(1):2712736. doi: 10.1080/21505594.2026.2712736 (PMC13483470; doi:10.1080/21505594.2026.2712736)
Supplement: Supplementary file 2 [file KVIR_A_2712736_SM3660.docx]

**Table S1** Main characteristics of the included studies in bovine.

| **Study ID** | **Country** | **Test method** | **Event** | **N** | **Positive rate** |
| --- | --- | --- | --- | --- | --- |
| Peng Z.H.et.al.(2020)[1] | China | RT-qPCR | 54 | 72 | 0.7500 |
| Peng H.et.al.(2020)[2] | China | RT-qPCR | 3 | 47 | 0.0638 |
| Sun F.Y.et.al.(2023)[3] | China | PCR | 97 | 462 | 0.2100 |
| Shen F.R.et.al.(2016)[4] | China | RT-qPCR | 20 | 39 | 0.5128 |
| Hu L.J.et.al.(2020)[5] | China | ELISA | 596 | 603 | 0.9884 |
| Liu H.Y.et.al.(2009)[6] | China | ELISA | 167 | 256 | 0.6523 |
| Luo Y.H.et.al.(2023)[7] | China | PCR | 6 | 422 | 0.0142 |
| Shi Q.et.al.(2014)[8] | China | ELISA | 25 | 25 | 1.0000 |
| Li Z.et.al.(2012)[9] | China | PCR | 7 | 42 | 0.1667 |
| Wang W.J.et.al.(2020)[10] | China | PCR | 19 | 127 | 0.1496 |
| Wei S.C.et.al.(2013)[11] | China | RT-qPCR | 10 | 62 | 0.1613 |
| Lang L.M.et.al.(2023)[12] | China | ELISA | 4 | 6 | 0.6667 |
| Ji S.et.al.(2023)[13] | China | PCR | 9 | 222 | 0.0405 |
| Shi X.J.et.al.(2023)[14] | China | PCR | 80 | 350 | 0.2286 |
| Zhang M.Y.et.al.(2023)[15] | China | RT-qPCR | 8 | 230 | 0.0348 |
| Wei S.T.et.al.(2021)[16] | China | PCR | 31 | 186 | 0.1667 |
| Pu P.et.al.(2024)[17] | China | RT-qPCR | 27 | 132 | 0.2045 |
| Sun Y.J.et.al.(2021)[18] | China | RT-qPCR | 8 | 29 | 0.2759 |
| Sun S.Q.(2024)[19] | China | RT-qPCR | 19 | 114 | 0.1667 |
| Liu B.X.(2021)[20] | China | PCR | 29 | 804 | 0.0361 |
| Tan S.et.al.(2020)[21] | China | RT-qPCR | 109 | 153 | 0.7124 |
| Yu Y.et.al.(2021)[22] | China | PCR | 23 | 86 | 0.2674 |
| Li C.L.et.al. (2022)[23] | China | ELISA | 54 | 296 | 0.1824 |
| Guo, Z.et.al.(2019)[24] | China | PCR | 15 | 98 | 0.1531 |
| Mekata,et.al.(2020)[25] | Japan | PCR | 85 | 455 | 0.1868 |
| Park.et.al.(2007)[26] | Korea | PCR | 1 | 645 | 0.0016 |
| Zhu Q.H.et.al.(2022)[27] | China | PCR | 11 | 160 | 0.0688 |
| Li S.Y. et.al.(2024)[28] | China | PCR | 560 | 1646 | 0.3402 |
| Lotfollahzadeh et.al.(2020)[29] | Iran | ELISA | 14 | 194 | 0.0722 |
| Debelo et.al.(2021)[30] | Ethiopia | ELISA | 1 | 110 | 0.0091 |
| Kim et.al.(2022)[31] | Korea | PCR | 50 | 846 | 0.0591 |
| Gomez et.al.(2017)[32] | Canada | PCR | 157 | 286 | 0.5490 |
| Zhang M. et.al.(2021)[33] | Canada | PCR | 140 | 310 | 0.4516 |
| Lee et.al.(2019)[34] | Korea | PCR | 13 | 207 | 0.0628 |
| Castells et.al.(2019)[35] | Uruguay | PCR | 64 | 824 | 0.0777 |
| Martinez (2012)[36] | Cuba | PCR | 30 | 136 | 0.2206 |
| Zhu Q.H. et.al.(2022)[37] | China | PCR | 158 | 1130 | 0.1398 |
| Glotov et.al.(2023)[38] | Russia | PCR | 395 | 1508 | 0.2619 |
| Park et.al.(2006)[39] | Korea | ELISA | 8 | 75 | 0.1067 |
| Wei X.J. et.al.(2021)[40] | China | PCR | 12 | 69 | 0.1739 |
| Cao X.W. et.al. (2024)[41] | China | PCR | 4 | 9 | 0.4444 |
| Chang L.Y.et.al.(2021)[42] | China | PCR | 142 | 788 | 0.1802 |
| Deng F.et.al.(2021)[43] | China | RT-qPCR | 65 | 387 | 0.1680 |
| Gao H.et.al.(2022)[44] | China | RT-qPCR | 131 | 1069 | 0.1225 |
| Huang J.et.al.(2023)[45] | China | RT-qPCR | 204 | 279 | 0.7312 |
| Jia K.W.(2024)[46] | China | PCR | 67 | 421 | 0.1591 |
| Kang T.S.et.al.(2024)[47] | China | RT-qPCR | 29 | 445 | 0.0652 |
| Li S.Y.et.al.(2024)[48] | China | PCR | 46 | 323 | 0.1424 |
| Luo J.D.et.al.(2024)[49] | China | RT-qPCR | 235 | 1819 | 0.1292 |
| Ma Z.X.(2017)[50] | China | ELISA | 119 | 326 | 0.3650 |
| Wang M.J.et.al.(2023)[51] | China | PCR | 156 | 652 | 0.2393 |
| Zhang S.H.et.al.(2022)[52] | China | PCR | 51 | 357 | 0.1429 |
| Zhao X.L.et.al.(2020)[53] | China | ELISA | 75 | 210 | 0.3571 |
| Keha ABI (2019)[54] | China | PCR | 36 | 190 | 0.1895 |
| Chen K.J.et.al.(2022)[55] | China | PCR & ELISA | 353 | 1080 | 0.3269 |
| Cui S.W.et.al.(2024)[56] | China | PCR | 38 | 334 | 0.1138 |
| Cui X.(2019)[57] | China | ELISA | 31 | 325 | 0.0954 |
| Guo S.H.(2021)[58] | China | PCR | 40 | 424 | 0.0943 |
| Guo S.H.et.al.(2022)[59] | China | PCR | 10 | 106 | 0.0943 |
| Han Y.X.et.al.(2024)[60] | China | PCR | 8 | 10 | 0.8000 |
| He Q.F.et.al.(2019)[61] | China | PCR | 232 | 336 | 0.6905 |
| Huang Q.et.al.(2024)[62] | China | PCR | 9 | 74 | 0.1216 |
| Xiong X.X.(2022)[63] | China | PCR | 11 | 246 | 0.0447 |
| Zhou J.(2018)[64] | China | RT-qPCR | 98 | 130 | 0.7538 |
| Zhang L.et.al.(2020)[65] | China | PCR | 32 | 362 | 0.0884 |
| Jiang J.(2007)[66] | China | PCR & ELISA | 152 | 2368 | 0.0642 |
| Ling W.X.(2023)[67] | China | PCR & ELISA | 239 | 901 | 0.2653 |
| Liu H.Y.et.al.(2009)[6] | China | ELISA | 244 | 256 | 0.9531 |
| Liu Y.(2020)[68] | China | ELISA | 41 | 57 | 0.7193 |
| Peng L.(2022)[69] | China | ELISA | 46 | 178 | 0.2584 |
| Shi J.C.(2023)[70] | China | PCR | 22 | 204 | 0.1078 |
| Wang C.N.(2024)[71] | China | PCR | 242 | 1100 | 0.2200 |
| Zhang K.et.al.(2015)[72] | China | PCR | 12 | 71 | 0.1690 |

[1]	Peng ZH, Han QY, Cui MJ, Fan TL, Liu Y, Wang MJ, et al. Development and application of a duplex TaqMan real-time fluorescent quantitative PCR assay for detection of bovine rotavirus and bovine coronavirus[J]. Chinese Journal of Preventive Veterinary Medicine, 2020, 42(10): 1009-1013.

[2]	Peng H, Wu CL, Li J, Li CT, Tao L, Bai HL, et al. Detection and analysis of coronavirus in bovine diarrhea cases in some areas of Guangxi[J]. Progress in Veterinary Medicine, 2020, 41(11): 122-124. doi:10.16437/j.cnki.1007-5038.2020.11.024

[3]	Sun FY, Ye JF, Wei Y, Wang ZX, Zhang JY, Bing LY, et al. Investigation and analysis of bovine coronavirus infection status in beef cattle in Jilin Province[J]. Acta Veterinaria et Zootechnica Sinica, 2023, 54(02): 673-682.

[4]	Shen FR, Yang JL, Zhao GM, ZhuT, Cheng KH, Wang HM, et al. Development and preliminary application of a SYBR Green Ⅰ real-time fluorescent quantitative PCR assay for detecting bovine coronavirus[J]. Chinese Journal of Veterinary Medicine, 2016, 52(06): 22-24+27.

[5]	Hu LJ, Meng Y, Zhou YL, Jia WQ, Zhai HR, Wu R, et al. Development and application of an indirect ELISA for the detection of bovine coronavirus antibodies[J]. Microbiology China, 2020, 47(1): 330-338.doi: 10.13344/j.microbiol.china.190422

[6]	Liu HY, Sun LX, Wang JY, Liu SY, Wang H, Yu LY, et al. Development of an indirect ELISA for detecting bovine coronavirus based on recombinant N protein[J]. Chinese Journal of Preventive Veterinary Medicine, 2009, 31(8): 618-622.

[7]	Luo YH, Liu HH, Li DZ, Zhang GX, Qiao CP, Pan Y, et al. Epidemiological investigation and genetic variation analysis of bovine enterovirus and bovine coronavirus in Hechi area of Guangxi[J]. Chinese Journal of Veterinary Medicine, 2023, 59(07): 32-39.

[8]	Shi Q, Yuan LG, Pu JW, Li J, Xu JJ, Sha L, et al. Serological monitoring and investigation of six diseases including brucellosis in Tianshan yaks[J]. China Dairy Cattle, 2014(16): 31-32.

[9]	Li Z, Tian B, Li M S, Ping L. Development and preliminary application of a reverse transcription PCR assay for the rapid detection of bovine viral diarrhea virus[J]. Journal of Northwest Minzu University, 2012, 33(1): 47-52.doi: 10.14084/j.cnki.cn62-1188/n.2012.01.007

[10]	Wang WJ, Xu ZX, Lan YL, Shi ZH. Development and preliminary application of a multiplex PCR assay for the detection of bovine coronavirus, bovine norovirus and bovine kobuvirus[J]. Chinese Journal of Veterinary Science, 2020, 40(7): 1306-1310,1355.doi: 10.16303/j.cnki.1005-4545.2020.07.08

[11]	Wei SC, Gong ZD, Che TJ, Tian FL. Development of a fluorescent quantitative RT-PCR assay for the VP7 gene of bovine rotavirus[J]. Chinese Journal of Preventive Veterinary Medicine, 2013, 35(2): 151-154.

[12]	Lang LM, Chen FY, Fang JY, Zhang LX, You Y, Feng YJ, et al. Diagnosis and treatment of calf diarrhea caused by mixed infection of coronavirus and rotavirus[J]. China Cattle Science, 2023, 49(1): 90-92.

[13]	Ji S, Peng YN, Ye Z, Wang SS, Wang ZY. Development and application of a one-step quadruple RT-PCR assay for the differential detection of bovine viral diarrhea virus, bovine enterovirus, bovine rotavirus and bovine coronavirus[J]. Chinese Journal of Animal Infectious Diseases, 2023, 31(5): 120-127.doi: 10.19958/j.cnki.cn31-2031/s.20210525.001

[14]	Shi XJ, Xie MY, Zhang YY, Guo Y, Sa RL, Chen KJ, et al. Epidemiological investigation of BCoV in some large-scale cattle farms in Tongliao City, Inner Mongolia Autonomous Region[J]. Veterinary Orientation, 2023(2): 20-23.

[15]	Zhang MY, Zhong LH, Han YY, Liu YM, Li MX, Wang M, et al. Development and application of a multiplex real-time fluorescent quantitative PCR assay for the detection of major viruses causing bovine diarrhea[J]. Progress in Veterinary Medicine, 2023, 44(11): 26-32.doi: 10.16437/j.cnki.1007-5038.2023.11.018

[16]	Wei ST, Xie J, Xiong XX, Ma JR, Li RQ, Miao XP, et al. Development and application of a multiplex PCR assay for the detection of bovine respiratory viruses[J]. Chinese Journal of Veterinary Science, 2021, 41(11): 2126-2131.doi: 10.16303/j.cnki.1005-4545.2021.11.07

[17]	Pu P, Li CL, Zhang Q, Wu FX, Xu XG. Development and application of a TaqMan real-time fluorescent quantitative PCR assay for bovine coronavirus[J]. Progress in Veterinary Medicine, 2024, 45(02): 7-10.doi: 10.16437/j.cnki.1007-5038.2024.02.001

[18]	Sun YJ, Guan PY, Zhou WG, Xu XJ, Xi NN G, Yu JL. Development of a TaqMan triplex RT-qPCR assay for BVDV, BRV and BCoV[J]. China Animal Quarantine, 2021, 38(03): 99-106.

[19]	Sun SQ. Development and application of a TaqMan triplex RT-qPCR assay for BVDV, BRV and BCoV[D]. 2024.

[20]	Liu BX. Investigation on four diarrhea pathogens in meat calves in some areas of Hebei Province and establishment of a BVDV antibody detection method[D]. 2021.

[21]	Tan S, Tang C, He QF, Yue H. Development and preliminary application of a TaqMan real-time fluorescent quantitative RT-PCR assay for bovine coronavirus[J]. Chinese Journal of Preventive Veterinary Medicine, 2020, 42(9): 918-923.

[22]	Yu Y, Zhou J, Zhang B. Detection of six diarrhea-related viruses in dairy cows in Xinjiang Uygur Autonomous Region[J]. Progress in Veterinary Medicine, 2021, 42(10): 139-142.doi: 10.16437/j.cnki.1007-5038.2021.10.027

[23]	Li CL, Wu FX, Xu HL, Xu XG, Zhang Q. Development and application of an indirect ELISA for the detection of bovine coronavirus antibodies based on N protein[J]. Progress in Veterinary Medicine, 2022, 43(5): 1-5.doi: 10.16437/j.cnki.1007-5038.2022.05.013

[24]	Guo Z, He Q, Zhang B, Yue H, Tang C. Detection and molecular characteristics of neboviruses in dairy cows in China. The Journal of general virology. 2019;100(1):35-45.doi: 10.1099/jgv.0.001172

[25]	Mekata H, Hamabe S, Sudaryatma PE, Kobayashi I, Kanno T, Okabayashi T. Molecular epidemiological survey and phylogenetic analysis of bovine respiratory coronavirus in Japan from 2016 to 2018. The Journal of veterinary medical science. 2020;82(6):726-30.doi: 10.1292/jvms.19-0587

[26]	Park SI, Jeong C, Kim HH, Park SH, Park SJ, Hyun BH, et al. Molecular epidemiology of bovine noroviruses in South Korea. Veterinary microbiology. 2007;124(1-2):125-33.doi: 10.1016/j.vetmic.2007.03.010

[27]	Zhu Q, Su M, Li Z, Wang X, Qi S, Zhao F, et al. Epidemiological survey and genetic diversity of bovine coronavirus in Northeast China. Virus Research. 2022;308.doi: 10.1016/j.virusres.2021.198632

[28]	Li S, Huang J, Cai X, Mao L, Xie L, Wang F, et al. Prevalence and Evolutionary Characteristics of Bovine Coronavirus in China. Veterinary Sciences. 2024;11(6).doi: 10.3390/vetsci11060230

[29]	Lotfollahzadeh S, Madadgar O, Reza Mohebbi M, Reza Mokhber Dezfouli M, George Watson D. Bovine coronavirus in neonatal calf diarrhoea in Iran. Vet Med Sci. 2020;6(4):686-94.doi: 10.1002/vms3.277

[30]	Debelo M, Abdela H, Tesfaye A, Tiruneh A, Mekonnen G, Asefa Z, et al. Prevalence of Bovine Rotavirus and Coronavirus in Neonatal Calves in Dairy Farms of Addis Ababa, Ethiopia: Preliminary Study. Biomed Res Int. 2021;2021:5778455.doi: 10.1155/2021/5778455

[31]	Kim EM, Cho HC, Shin SU, Park J, Choi KS. Prevalence and genetic characterization of bovine coronavirus identified from diarrheic pre-weaned native Korean calves from 2019 to 2021. Infection, genetics and evolution : journal of molecular epidemiology and evolutionary genetics in infectious diseases. 2022;100:105263.doi: 10.1016/j.meegid.2022.105263

[32]	Gomez DE, Arroyo LG, Poljak Z, Viel L, Weese JS. Detection of Bovine Coronavirus in Healthy and Diarrheic Dairy Calves. J Vet Intern Med. 2017;31(6):1884-91.doi: 10.1111/jvim.14811

[33]	Zhang M, Hill JE, Alexander TW, Huang Y. The nasal viromes of cattle on arrival at western Canadian feedlots and their relationship to development of bovine respiratory disease. Transbound Emerg Dis. 2021;68(4):2209-18.doi: 10.1111/tbed.13873

[34]	Lee SH, Kim HY, Choi EW, Kim D. Causative agents and epidemiology of diarrhea in Korean native calves. J Vet Sci. 2019;20(6):e64.doi: 10.4142/jvs.2019.20.e64

[35]	Castells M, Giannitti F, Caffarena RD, Casaux ML, Schild C, Castells D, et al. Bovine coronavirus in Uruguay: genetic diversity, risk factors and transboundary introductions from neighboring countries. Arch Virol. 2019;164(11):2715-24.doi: 10.1007/s00705-019-04384-w

[36]	Martinez N, Brandao PE, de Souza SP, Barrera M, Santana N, Diaz de Arce H, et al. Molecular and phylogenetic analysis of bovine coronavirus based on the spike glycoprotein gene. Infection Genetics and Evolution. 2012;12(8):1870-8.doi: 10.1016/j.meegid.2012.05.007

[37]	Zhu Q, Su M, Li Z, Wang X, Qi S, Zhao F, et al. Epidemiological survey and genetic diversity of bovine coronavirus in Northeast China. Virus Research. 2022;308:198632. doi: 10.1016/j.virusres.2021.198632

[38]	Glotov AG, Nefedchenko AV, Yuzhakov AG, Koteneva SV, Glotova TI, Komina AK, et al. Genetic diversity of Siberian bovine coronavirus isolates (Coronaviridae: Coronavirinae: Betacoronavirus-1: Bovine-Like coronaviruses). Voprosy virusologii. 2023;67(6):465-74.doi: 10.36233/0507-4088-141

[39]	Park S-J, Jeong C, Yoon S-S, Choy HE, Saif LJ, Park S-H, et al. Detection and characterization of bovine coronaviruses in fecal specimens of adult cattle with diarrhea during the warmer seasons. Journal of Clinical Microbiology. 2006;44(9):3178-88.doi: 10.1128/jcm.02667-05

[40]	Wei X, Wang W, Dong Z, Cheng F, Zhou X, Li B, et al. Detection of Infectious Agents Causing Neonatal Calf Diarrhea on Two Large Dairy Farms in Yangxin County, Shandong Province, China. Frontiers in Veterinary Science. 2021;7.doi: 10.3389/fvets.2020.589126

[41]	Cao XW, Fu XYN, Chen Y, Gao HY, Wang D, Jiang S. Study on calf diarrhea caused by mixed infection of bovine coronavirus, bovine rotavirus and pathogenic Escherichia coli[J]. China Cattle Science, 2024, 50(2): 51-58,62. doi: 10.3969/j.issn.1001-9111.2024.02.011

[42]	Chang LY, Liu ZY, Qin JH, Zhao YL. Epidemiological investigation of viral diarrhea in dairy calves in Tangshan City, Hebei Province[J]. China Animal Husbandry & Veterinary Medicine, 2021, 48(11): 4213-4219.doi: 10.16431/j.cnki.1671-7236.2021.11.031

[43]	Deng F, Chen DS, Chen B, Shao L, Chen D, Zhou LY, et al. Epidemiological investigation of bovine coronavirus infection in diarrheic cattle herds in some areas of Sichuan Province in 2020[J]. China Animal Quarantine, 2021, 38(09): 13-16.

[44]	Gao H, Li XC, Liu Y, Yuan Y, Zhang XY, Ni B, et al. Development and application of a real-time fluorescent quantitative RT-PCR assay for bovine coronavirus[J]. Heilongjiang Animal Science and Veterinary Medicine, 2022(06): 86-91.doi: 10.13881/j.cnki.hljxmsy.2021.09.0301

[45]	Huang J, Li SY, Xie LL, Zhou D, Yang R, Wang S, et al. Development of a TaqMan real-time fluorescent detection assay for bovine coronavirus and epidemiological investigation in some areas of China[J]. Jiangsu Agricultural Sciences, 2023, 51(17): 29-33.

[46]	Jia KW. Epidemiological investigation of BVDV, BCoV, BNoV and BToV in calves in intensive cattle farms in Xinjiang and isolation and identification of BCoV[D]. 2023.

[47]	Kang TS, Liu Y, Hu YF, Li BW, Wang YB, Wang SJ, et al. Development and application of a one-step triplex real-time fluorescent PCR assay for bovine coronavirus, bovine enterovirus and bovine viral diarrhea virus[J]. Chinese Veterinary Science, 2024, 54(4): 472-478.doi: 10.16656/j.issn.1673-4696.2024.0070

[48]	Li SY, Fu XC, Yuan XS, Mao L, Cai XH, Sun XR, et al. Detection of major viral diarrhea pathogens in cattle in Langfang City, Hebei Province and evolutionary analysis of bovine coronavirus[J]. Acta Veterinaria et Zootechnica Sinica, 2024, 55(2): 649-659.

[49]	Luo JD, Zhang XY, Fang LL, Liu S, Zheng H, Wei R, et al. Genetic variation analysis of the HE gene of bovine coronavirus in some provinces of China[J]. Progress in Veterinary Medicine, 2024, 45(9): 81-87.doi: 10.3969/j.issn.1007-5038.2024.09.015

[50]	Ma ZX. Epidemiological investigation of bovine coronavirus in Qingfeng County, Henan Province[J]. China Dairy Industry, 2017(6): 65-66.

[51]	Wang MJ, Jiang Q, Ma XJ, Xia RY, Guo XP, Sun L, et al. Molecular epidemiological investigation of bovine coronavirus in Xinjiang Uygur Autonomous Region[J]. Acta Veterinaria et Zootechnica Sinica, 2023, 54(12): 5125-5133.

[52]	Zhang SH, Wang XW, Dai Q, Deng CS, Lu J Q, Yu SX, et al. Detection of bovine coronavirus in some areas of Yunnan Province and sequence analysis of its N gene[J]. China Animal Quarantine, 2022, 39(11): 35-40.

[53]	Zhao XL, Niu JQ, Ciren YJ, Ciren YZ, Suolang ZG, Wen DX, et al. Epidemiological investigation of BVDV, BCV and BRV in yak diarrhea viruses[J]. Plateau Agriculture, 2020, 4(3): 298-302.doi: 10.19707/j.cnki.jpa.2020.03.013

[54]	Keha ABI. Molecular detection, genomic characteristics of dairy cow coronavirus and development of an ELISA kit[D]. 2019.

[55]	Chen KJ, Xie MY, Guo Y, Shang HW, Chang JT, Gao DJ, et al. Epidemiological investigation of bovine coronavirus in some large-scale cattle farms in Inner Mongolia Autonomous Region[J]. Heilongjiang Animal Science and Veterinary Medicine, 2023(14): 77-81. doi: 10.13881/j.cnki.hljxmsy.2022.07.0236

[56]	Cui SW, Zhao QM, Yu K, Xue JQ, Sun RH, Li Y, et al. Epidemiological investigation of diarrhea-related pathogens in lactating dairy calf herds in some areas of Ningxia Hui Autonomous Region[J]. Acta Agriculturae Boreali-Occidentalis Sinica, 2024, 33(9): 1599-1613.

[57]	Cui X. Epidemiological investigation of rotavirus and coronavirus in some large-scale dairy farms in southern Xinjiang[D]. 2019.

[58]	Guo SH. Study on the diversity of intestinal microflora and fecal metabolome in diarrheic calves[D]. 2021.

[59]	Guo SH, Liang T, Cao C, Yu DB, Wang F, Jiang ZG, et al. Epidemiological investigation of neonatal calf diarrhea pathogens in some areas of Ningxia Hui Autonomous Region and genetic evolution analysis of major pathogens[J]. Acta Agriculturae Boreali-Occidentalis Sinica, 2022, 31(4): 413-424.

[60]	Han YX, Zhang JJ, Wang YY, Li QQ, Liu HH, Chen JJ, et al. Etiological identification of neonatal meat calf diarrhea in an outbreak[J]. Journal of Animal Husbandry and Veterinary Medicine Information, 2024(3): 33-36.

[61]	He QF, Tang C, Guo ZJ, Tan S, Zhang B, Yue H. Amplification, sequence analysis and virus isolation and identification of partial genes of yak-derived bovine coronavirus[J]. Acta Veterinaria et Zootechnica Sinica, 2019, 50(2): 343-353.

[62]	Huang Q, Yang B, Zhang YM, Liang H, Su S, Hu JJ, et al. Isolation, identification and N gene evolutionary analysis of calf coronavirus in southern Xinjiang[J]. Journal of Economic Animal Science,p: 1-6.

[63]	Xiong XX. Molecular virological investigation of calf diarrhea in Ningxia Hui Autonomous Region and preliminary analysis of calf fecal virome[D]. 2022.

[64]	Zhou J. Development and application of an isothermal insulated fluorescent RT-PCR kit for bovine viral diarrhea virus type 1[D]. 2018.

[65]	Zhang L, Zhang YF, Jiang RX, Ren YC, Zhang M, Chu HM, et al. Etiological detection of diarrhea-related viruses in dairy calves in dairy farms of Shandong Province from 2017 to 2018[J]. China Animal Quarantine, 2020, 37(6): 16-20.

[66]	Jiang J, Li J, Hu YQ, Qiu L, Xiong W, Wang QQ. Epidemiological investigation of animal coronaviruses in four provinces and municipalities including Shanghai[J]. Animal Husbandry & Veterinary Medicine, 2007(12): 50-52.

[67]	Ling W X. Epidemiological investigation of bovine coronavirus disease in some areas of Yunnan Province[D]. 2023.

[68]	Liu Y. Establishment and preliminary application of diagnostic methods for bovine coronavirus[D]. 2020.

[69]	Peng L. Detection of calf viral diarrhea pathogens and establishment of an ELISA method for bovine coronavirus[D]. 2022.

[70]	Shi JC. Epidemiological investigation and prevention and control of pathogens associated with bovine respiratory disease complex in a large beef cattle farm in Xinjiang[D]. 2023.

[71]	Wang C N. Development and preliminary application of a one-step triplex fluorescent quantitative PCR assay for BRV, BCoV and K99[D]. 2024.

[72]	Zhang K, Yan GQ, Wang JM, Yang MW, Ling C, Shi YX, et al. Molecular epidemiological investigation of coronaviruses causing calf diarrhea in some areas of northern Xinjiang[J] Chinese Veterinary Science. 2015;45(12):1270-6.doi: 10.16656/j.issn.1673-4696.2015.12.011
