# Supplementary material for: Epidemiological characteristics and structural-functional implications of spike protein variations in a bovine coronavirus isolate from diarrheic calves
Source: Virulence. 2026 Aug 17;17(1):2712736. doi: 10.1080/21505594.2026.2712736 (PMC13483470; doi:10.1080/21505594.2026.2712736)
Supplement: Supplementary file 3 [file KVIR_A_2712736_SM3664.docx]

**Supplementary Figure Legends**

Figure S1. Forest map of global bovine infection with BCoV.

Figure S2. Test for Publication Bias. (a) Funnel plot with pseudo 95% confidence interval for publication bias test. (b) Egger’s test for publication bias. (c) Trim and fill analysis and pseudo 95% confidence interval publication bias test.

Figure S3. Sensitivity analysis of bovine infection with BCoV.

Figure S4. Map of BCoV prevalence worldwide.

**Supplementary Table Legends**

Table S1. Main characteristics of the included studies in bovine.

Table S2. Normal distribution tests for normal rates and different transitions of articles hosted by bovine.

Table S3. Reference Strains for the BCoV Complete Genome Sequence.

Table S4. Reference Strains for the BCoV S Gene Sequence.

Table S5. Whole-genome nucleotide identity analysis of BCoV.

Table S6 Nucleotide identity analysis of the BCoV S gene.
