# Supplementary material for: Epidemiological characteristics and structural-functional implications of spike protein variations in a bovine coronavirus isolate from diarrheic calves
Source: Virulence. 2026 Aug 17;17(1):2712736. doi: 10.1080/21505594.2026.2712736 (PMC13483470; doi:10.1080/21505594.2026.2712736)
Supplement: Supplementary file 4 [file KVIR_A_2712736_SM3666.docx]

**Table S4** Reference Strains for the BCoV S Gene Sequence.

| **Strain ID** | **Virus species** | **Country** | **Year** |
| --- | --- | --- | --- |
| AY585228 | HCoV OC43 | USA | 2004 |
| KF169908 | BCoV | Sweden | 1992 |
| KF169922 | BCoV | Sweden | 2006 |
| KF169924 | BCoV | Sweden | 2013 |
| KF169940 | BCoV | Sweden | 2010 |
| KF169939 | BCoV | Sweden | 2010 |
| OR271252 | BCoV | Ireland | 2022 |
| OR271249 | BCoV | Ireland | 2022 |
| KF169933 | BCoV | Sweden | 2008 |
| KF169938 | BCoV | Sweden | 2010 |
| KF169937 | BCoV | Sweden | 2010 |
| KT318116 | BCoV | France | 2010 |
| KT318114 | BCoV | France | 2008 |
| KF169914 | BCoV | Denmark | 2003 |
| KT318112 | BCoV | France | 2005 |
| KT318124 | BCoV | France | 2004 |
| KT318113 | BCoV | France | 2007 |
| KT318123 | BCoV | France | 2014 |
| KT318120 | BCoV | France | 2013 |
| KT318119 | BCoV | France | 2013 |
| MG757144 | BCoV | France | 2014 |
| MG757143 | BCoV | France | 2014 |
| KT318121 | BCoV | France | 2013 |
| KT318122 | BCoV | France | 2014 |
| KT318118 | BCoV | France | 2013 |
| KT318117 | BCoV | France | 2012 |
| KF272919 | BCoV | Ireland | 2016 |
| KF169910 | BCoV | Sweden | 2002 |
| KF169909 | BCoV | Sweden | 2002 |
| KF169912 | BCoV | Sweden | 2002 |
| KF169911 | BCoV | Sweden | 2002 |
| KF169935 | BCoV | Sweden | 2009 |
| KF169934 | BCoV | Sweden | 2009 |
| KF169928 | BCoV | Sweden | 2007 |
| KF169921 | BCoV | Sweden | 2005 |
| KF169920 | BCoV | Sweden | 2005 |
| KF169923 | BCoV | Sweden | 2013 |
| KF169936 | BCoV | Sweden | 2013 |
| KF169930 | BCoV | Sweden | 2007 |
| KF169925 | BCoV | Sweden | 2007 |
| KF169932 | BCoV | Sweden | 2008 |
| KF169931 | BCoV | Sweden | 2008 |
| KF169926 | BCoV | Sweden | 2007 |
| KF169917 | BCoV | Denmark | 2005 |
| KF169919 | BCoV | Denmark | 2005 |
| KF169916 | BCoV | Denmark | 2005 |
| KF169918 | BCoV | Denmark | 2005 |
| KT318115 | BCoV | France | 2003 |
| KT318111 | BCoV | France | 2003 |
| KF169915 | BCoV | Denmark | 2003 |
| KF169913 | BCoV | Denmark | 2003 |
| MK046008 | BCoV | Viet Nam | 2019 |
| MK046007 | BCoV | Viet Nam | 2019 |
| MW521184 | BCoV | China | 2020 |
| MZ711352 | BCoV | China | 2020 |
| MZ711355 | BCoV | China | 2020 |
| MZ711353 | BCoV | China | 2020 |
| MZ711354 | BCoV | China | 2020 |
| DQ389658 | BCoV | South_Korea | 2006 |
| DQ389656 | BCoV | South_Korea | 2006 |
| EU686689 | BCoV | South_Korea | 2009 |
| EU401986 | BCoV | South_Korea | 2009 |
| OP186320 | BCoV | South_Korea | 2017 |
| HM573329 | BCoV | South_Korea | 2010 |
| HM573326 | BCoV | South_Korea | 2010 |
| OP186328 | BCoV | South_Korea | 2018 |
| OP186327 | BCoV | South_Korea | 2018 |
| OP186324 | BCoV | South_Korea | 2018 |
| OP186323 | BCoV | South_Korea | 2017 |
| OP186326 | BCoV | South_Korea | 2017 |
| OP186325 | BCoV | South_Korea | 2017 |
| OP186313 | BCoV | South_Korea | 2017 |
| OP186317 | BCoV | South_Korea | 2017 |
| OR909657 | BCoV | South_Korea | 2017 |
| OR909656 | BCoV | South_Korea | 2017 |
| OR909655 | BCoV | South_Korea | 2017 |
| OP186318 | BCoV | South_Korea | 2017 |
| OR909661 | BCoV | South_Korea | 2017 |
| OR909660 | BCoV | South_Korea | 2017 |
| OR909659 | BCoV | South_Korea | 2017 |
| OR909658 | BCoV | South_Korea | 2017 |
| OR909665 | BCoV | South_Korea | 2017 |
| OR909662 | BCoV | South_Korea | 2017 |
| OP186314 | BCoV | South_Korea | 2017 |
| OP186315 | BCoV | South_Korea | 2017 |
| OP186322 | BCoV | South_Korea | 2017 |
| OP186321 | BCoV | South_Korea | 2017 |
| OP186319 | BCoV | South_Korea | 2017 |
| OP186316 | BCoV | South_Korea | 2017 |
| DQ389655 | BCoV | South_Korea | 2006 |
| DQ389653 | BCoV | South_Korea | 2006 |
| DQ389633 | BCoV | South_Korea | 2006 |
| DQ389654 | BCoV | South_Korea | 2006 |
| DQ389634 | BCoV | South_Korea | 2006 |
| DQ389635 | BCoV | South_Korea | 2006 |
| DQ389632 | BCoV | South_Korea | 2006 |
| DQ389636 | BCoV | South_Korea | 2006 |
| DQ389639 | BCoV | South_Korea | 2006 |
| DQ389641 | BCoV | South_Korea | 2006 |
| DQ389637 | BCoV | South_Korea | 2006 |
| DQ389640 | BCoV | South_Korea | 2006 |
| DQ389638 | BCoV | South_Korea | 2006 |
| DQ389659 | BCoV | South_Korea | 2006 |
| DQ389652 | BCoV | South_Korea | 2006 |
| DQ389657 | BCoV | South_Korea | 2006 |
| DQ389660 | BCoV | South_Korea | 2006 |
| AY935641 | BCoV | South_Korea | 2005 |
| AY935640 | BCoV | South_Korea | 2005 |
| AY935646 | BCoV | South_Korea | 2005 |
| AY935645 | BCoV | South_Korea | 2005 |
| AY935637 | BCoV | South_Korea | 2005 |
| AY935642 | BCoV | South_Korea | 2005 |
| AY935638 | BCoV | South_Korea | 2005 |
| AY935644 | BCoV | South_Korea | 2005 |
| AY935639 | BCoV | South_Korea | 2005 |
| MW521193 | BCoV | China | 2020 |
| MK046004 | BCoV | Viet Nam | 2019 |
| OR136878 | BCoV | China | 2021 |
| MK095184 | BCoV | China | 2018 |
| MK095185 | BCoV | China | 2018 |
| MK095176 | BCoV | China | 2017 |
| MK046011 | BCoV | Viet Nam | 2019 |
| MK046009 | BCoV | Viet Nam | 2019 |
| MH203067 | BCoV | Viet Nam | 2017 |
| MH203066 | BCoV | Viet Nam | 2017 |
| MK046003 | BCoV | Viet Nam | 2019 |
| MH197039 | BCoV | Viet Nam | 2017 |
| MH203064 | BCoV | Viet Nam | 2017 |
| MH197038 | BCoV | Viet Nam | 2017 |
| MH197037 | BCoV | Viet Nam | 2017 |
| MH203065 | BCoV | Viet Nam | 2017 |
| MK046010 | BCoV | Viet Nam | 2019 |
| MK046006 | BCoV | Viet Nam | 2019 |
| MK046005 | BCoV | Viet Nam | 2019 |
| PQ243295 | BCoV | China | 2024 |
| PQ243294 | BCoV | China | 2024 |
| MZ711360 | BCoV | China | 2021 |
| MZ711349 | BCoV | China | 2020 |
| MK095178 | BCoV | China | 2018 |
| MK095177 | BCoV | China | 2018 |
| MK095186 | BCoV | China | 2018 |
| MK095175 | BCoV | China | 2017 |
| MK095174 | BCoV | China | 2017 |
| MW521188 | BCoV | China | 2020 |
| OR947445 | BCoV | China | 2020 |
| MT975571 | BCoV | China | 2018 |
| MN982181 | BCoV | China | 2018 |
| MN982178 | BCoV | China | 2018 |
| MN982176 | BCoV | China | 2018 |
| MN982175 | BCoV | China | 2018 |
| MN982173 | BCoV | China | 2018 |
| MN982174 | BCoV | China | 2018 |
| MW521194 | BCoV | China | 2020 |
| MW711292 | BCoV | China | 2019 |
| MW711293 | BCoV | China | 2019 |
| MW711289 | BCoV | China | 2019 |
| MW521185 | BCoV | China | 2020 |
| OL456213 | BCoV | China | 2021 |
| MZ711347 | BCoV | China | 2020 |
| MZ711346 | BCoV | China | 2020 |
| MW521177 | BCoV | China | 2020 |
| MW711301 | BCoV | China | 2020 |
| MN982177 | BCoV | China | 2018 |
| MN982179 | BCoV | China | 2018 |
| MN982167 | BCoV | China | 2018 |
| MN982169 | BCoV | China | 2018 |
| MN982168 | BCoV | China | 2018 |
| MN982171 | BCoV | China | 2018 |
| MN982166 | BCoV | China | 2018 |
| MW711297 | BCoV | China | 2020 |
| MW711296 | BCoV | China | 2020 |
| MW521179 | BCoV | China | 2020 |
| MW521181 | BCoV | China | 2020 |
| MW521183 | BCoV | China | 2020 |
| MW521182 | BCoV | China | 2020 |
| MW521178 | BCoV | China | 2020 |
| MW521180 | BCoV | China | 2020 |
| MK095182 | BCoV | China | 2018 |
| MK095181 | BCoV | China | 2018 |
| MK095183 | BCoV | China | 2018 |
| MK095179 | BCoV | China | 2018 |
| OR947450 | BCoV | China | 2022 |
| OR947449 | BCoV | China | 2022 |
| OR947447 | BCoV | China | 2022 |
| OR947448 | BCoV | China | 2022 |
| OR947446 | BCoV | China | 2021 |
| MN982170 | BCoV | China | 2018 |
| OR947451 | BCoV | China | 2022 |
| PQ110069 | BCoV | China | 2024 |
| OR124732 | BCoV | China | 2022 |
| MW521187 | BCoV | China | 2020 |
| MW521186 | BCoV | China | 2020 |
| MZ711348 | BCoV | China | 2020 |
| MZ711358 | BCoV | China | 2021 |
| MW521192 | BCoV | China | 2020 |
| MW521191 | BCoV | China | 2020 |
| MW521190 | BCoV | China | 2020 |
| MW521189 | BCoV | China | 2020 |
| MZ603735 | BCoV | China | 2021 |
| MT975570 | BCoV | China | 2019 |
| PQ243293 | BCoV | China | 2024 |
| MW711294 | BCoV | China | 2020 |
| MW711291 | BCoV | China | 2019 |
| MK903506 | BCoV | China | 2019 |
| OR947455 | BCoV | China | 2022 |
| OR947454 | BCoV | China | 2022 |
| OR947453 | BCoV | China | 2022 |
| OR947452 | BCoV | China | 2022 |
| MZ711344 | BCoV | China | 2020 |
| MW521176 | BCoV | China | 2020 |
| MZ711345 | BCoV | China | 2020 |
| OR947444 | BCoV | China | 2020 |
| MZ711351 | BCoV | China | 2020 |
| MZ711350 | BCoV | China | 2020 |
| MW711290 | BCoV | China | 2019 |
| MW711304 | BCoV | China | 2020 |
| MW711298 | BCoV | China | 2020 |
| MW711295 | BCoV | China | 2020 |
| MW521175 | BCoV | China | 2020 |
| MZ711359 | BCoV | China | 2021 |
| MW711303 | BCoV | China | 2020 |
| MW711300 | BCoV | China | 2020 |
| MZ711357 | BCoV | China | 2020 |
| MZ711356 | BCoV | China | 2020 |
| MW711302 | BCoV | China | 2020 |
| MW711299 | BCoV | China | 2020 |
| OR464041 | BCoV | South_Korea | 2022 |
| EU401989 | BCoV | South_Korea | 2008 |
| EU401988 | BCoV | South_Korea | 2009 |
| EU401987 | BCoV | South_Korea | 2009 |
| OL990398 | BCoV | India | 2021 |
| OL960027 | BCoV | India | 2021 |
| DDFX98 | BCoV | China | 2024 |
| U00735 | BCoV | USA | 1972 |
| EF193075 | BCoV | Germany | 2006 |
| AF220295 | BCoV | Canada | 1972 |
