# Supplementary material for: Epidemiological characteristics and structural-functional implications of spike protein variations in a bovine coronavirus isolate from diarrheic calves
Source: Virulence. 2026 Aug 17;17(1):2712736. doi: 10.1080/21505594.2026.2712736 (PMC13483470; doi:10.1080/21505594.2026.2712736)
Supplement: Supplementary file 5 [file KVIR_A_2712736_SM3669.docx]

**Table S3** Reference Strains for the BCoV Complete Genome Sequence.

| **Strain ID** | **Virus species** | **Country** | **Year** |
| --- | --- | --- | --- |
| AY585228 | HCoV _OC43 | USA | 2004 |
| DDFX98 | BCoV | China | 2024 |
| PV335251 | BCoV | China | 2021 |
| PQ118397 | BCoV | USA | 2024 |
| PP235951 | BCoV | China | 2023 |
| PP599028 | BCoV | China | 2023 |
| PQ588962 | BCoV | China | 2024 |
| PQ313104 | BCoV | China | 2023 |
| LC830477 | BCoV | Japan | 2022 |
| LC830476 | BCoV | Japan | 2022 |
| OR502444 | BCoV | USA | 2023 |
| OR502443 | BCoV | USA | 2023 |
| OR502442 | BCoV | USA | 2021 |
| OR502441 | BCoV | USA | 2021 |
| OR502440 | BCoV | USA | 2021 |
| PP352170 | BCoV | China | 2020 |
| PP101629 | BCoV | China | 2023 |
| OR750853 | BCoV | China | 2023 |
| OR753439 | BCoV | China | 2023 |
| OR621178 | BCoV | China | 2022 |
| OR621177 | BCoV | China | 2022 |
| OR621176 | BCoV | China | 2022 |
| OR621175 | BCoV | China | 2022 |
| OR621174 | BCoV | China | 2022 |
| OR454204 | BCoV | China | 2023 |
| OR612027 | BCoV | China | 2022 |
| OR612026 | BCoV | China | 2022 |
| OR612025 | BCoV | China | 2022 |
| OR612024 | BCoV | China | 2022 |
| OR612023 | BCoV | China | 2022 |
| OR612022 | BCoV | China | 2022 |
| OR612021 | BCoV | China | 2022 |
| OR612020 | BCoV | China | 2022 |
| OR088593 | BCoV | China | 2023 |
| OR077317 | BCoV | China | 2021 |
| OR077316 | BCoV | China | 2022 |
| OR077315 | BCoV | China | 2022 |
| OR077314 | BCoV | China | 2021 |
| OR077313 | BCoV | China | 2022 |
| OR077312 | BCoV | China | 2023 |
| OR077311 | BCoV | China | 2023 |
| OR077310 | BCoV | China | 2023 |
| OR077309 | BCoV | China | 2023 |
| OR077308 | BCoV | China | 2023 |
| OR077307 | BCoV | China | 2023 |
| OR077306 | BCoV | China | 2022 |
| ON544073 | BCoV | China | 2021 |
| ON544072 | BCoV | China | 2021 |
| OQ718861 | BCoV | China | 2022 |
| OP004056 | BCoV | USA | 2022 |
| OQ507475 | BCoV | Norway | 2012 |
| OP924545 | BCoV | China | 2022 |
| OP866729 | BCoV | China | 2021 |
| OP866728 | BCoV | China | 2021 |
| OP866727 | BCoV | China | 2021 |
| OP866726 | BCoV | China | 2021 |
| OP296992 | BCoV | China | 2017 |
| ON142320 | BCoV | China | 2021 |
| ON142319 | BCoV | China | 2021 |
| ON142318 | BCoV | China | 2021 |
| ON142317 | BCoV | China | 2021 |
| ON142316 | BCoV | China | 2021 |
| ON142315 | BCoV | China | 2021 |
| ON792964 | BCoV | Ireland | 2020 |
| ON792962 | BCoV | Ireland | 2019 |
| ON792961 | BCoV | Ireland | 2019 |
| ON792960 | BCoV | Ireland | 2019 |
| ON792959 | BCoV | Ireland | 2019 |
| ON792956 | BCoV | Ireland | 2019 |
| ON792955 | BCoV | Ireland | 2019 |
| ON792954 | BCoV | Ireland | 2019 |
| ON792953 | BCoV | Ireland | 2019 |
| ON792952 | BCoV | Ireland | 2019 |
| ON792951 | BCoV | Ireland | 2019 |
| ON792950 | BCoV | Ireland | 2019 |
| ON792949 | BCoV | Ireland | 2019 |
| ON792948 | BCoV | Ireland | 2019 |
| ON792947 | BCoV | Ireland | 2019 |
| ON792946 | BCoV | Ireland | 2019 |
| ON792945 | BCoV | Ireland | 2019 |
| ON792943 | BCoV | Ireland | 2019 |
| ON792942 | BCoV | Ireland | 2019 |
| ON792941 | BCoV | Ireland | 2019 |
| ON093194 | BCoV | China | 2021 |
| ON146444 | BCoV | USA | 2014 |
| LC642814 | BCoV | Japan | 2020 |
| MW711287 | BCoV | China | 2020 |
| MN982199 | BCoV | China | 2018 |
| MN982198 | BCoV | China | 2018 |
| NC_003045 | BCoV | USA | 2001 |
| LC494178 | BCoV | Japan | 2016 |
| LC494177 | BCoV | Japan | 2008 |
| LC494176 | BCoV | Japan | 2016 |
| LC494175 | BCoV | Japan | 2016 |
| LC494174 | BCoV | Japan | 2008 |
| LC494173 | BCoV | Japan | 2008 |
| LC494172 | BCoV | Japan | 2007 |
| LC494171 | BCoV | Japan | 2010 |
| LC494170 | BCoV | Japan | 2010 |
| LC494169 | BCoV | Japan | 2009 |
| LC494168 | BCoV | Japan | 2009 |
| LC494167 | BCoV | Japan | 2008 |
| LC494166 | BCoV | Japan | 2008 |
| LC494165 | BCoV | Japan | 2008 |
| LC494164 | BCoV | Japan | 2006 |
| LC494162 | BCoV | Japan | 2006 |
| LC494161 | BCoV | Japan | 2007 |
| LC494160 | BCoV | Japan | 2007 |
| LC494159 | BCoV | Japan | 2017 |
| LC494158 | BCoV | Japan | 2016 |
| LC494157 | BCoV | Japan | 2015 |
| LC494156 | BCoV | Japan | 2014 |
| LC494155 | BCoV | Japan | 2014 |
| LC494154 | BCoV | Japan | 2014 |
| LC494153 | BCoV | Japan | 2017 |
| LC494152 | BCoV | Japan | 2017 |
| LC494151 | BCoV | Japan | 2017 |
| LC494150 | BCoV | Japan | 2017 |
| LC494149 | BCoV | Japan | 2016 |
| LC494148 | BCoV | Japan | 2016 |
| LC494147 | BCoV | Japan | 2016 |
| LC494146 | BCoV | Japan | 2016 |
| LC494145 | BCoV | Japan | 2016 |
| LC494144 | BCoV | Japan | 2016 |
| LC494143 | BCoV | Japan | 2016 |
| LC494142 | BCoV | Japan | 2016 |
| LC494141 | BCoV | Japan | 2016 |
| LC494140 | BCoV | Japan | 2015 |
| LC494139 | BCoV | Japan | 2015 |
| LC494138 | BCoV | Japan | 2015 |
| LC494137 | BCoV | Japan | 2013 |
| LC494136 | BCoV | Japan | 2012 |
| LC494135 | BCoV | Japan | 2012 |
| LC494134 | BCoV | Japan | 2012 |
| LC494133 | BCoV | Japan | 2011 |
| LC494132 | BCoV | Japan | 2011 |
| LC494131 | BCoV | Japan | 2011 |
| LC494130 | BCoV | Japan | 2010 |
| LC494128 | BCoV | Japan | 2012 |
| LC494127 | BCoV | Japan | 2012 |
| LC494126 | BCoV | Japan | 2016 |
| LC494192 | BCoV | Japan | 2017 |
| LC494191 | BCoV | Japan | 2017 |
| LC494189 | BCoV | Japan | 2017 |
| LC494188 | BCoV | Japan | 2017 |
| LC494187 | BCoV | Japan | 2017 |
| LC494186 | BCoV | Japan | 2017 |
| LC494185 | BCoV | Japan | 2017 |
| LC494184 | BCoV | Japan | 2017 |
| LC494183 | BCoV | Japan | 2017 |
| LC494182 | BCoV | Japan | 2016 |
| LC494180 | BCoV | Japan | 2016 |
| LC494179 | BCoV | Japan | 2016 |
| MG757141 | BCoV | France | 2014 |
| MG757140 | BCoV | France | 2014 |
| MG757139 | BCoV | France | 2014 |
| MG757138 | BCoV | France | 2014 |
| MH043955 | BCoV | USA | 2016 |
| MH043954 | BCoV | USA | 2017 |
| MH043953 | BCoV | USA | 2017 |
| MH043952 | BCoV | USA | 2017 |
| EF424620 | BCoV | USA | 2007 |
| EF424619 | BCoV | USA | 2007 |
| EF424618 | BCoV | USA | 2007 |
| EF424617 | BCoV | USA | 2007 |
| EF424616 | BCoV | USA | 2007 |
| EF424615 | BCoV | USA | 2007 |
| KU886219 | BCoV | China | 2015 |
| AF391542 | BCoV | USA | 2001 |
| AF220295 | BCoV | USA | 1972 |
| AF391541 | BCoV | USA | 2001 |
| DQ811784 | BCoV | USA | 1983 |
| FJ938064 | BCoV | USA | 2000 |
| FJ938063 | BCoV | USA | 1996 |
| U00735 | BCoV | USA | 1972 |
| DQ915164 | BCoV | USA | 2006 |
